# Supplementary figures and images for: Capsular polysaccharide restrains type VI secretion in Acinetobacter baumannii
Source: eLife. 2025 Jan 3;14:e101032. doi: 10.7554/eLife.101032 (PMC11731876; doi:10.7554/eLife.101032)

Figure 1A – source data-annotated

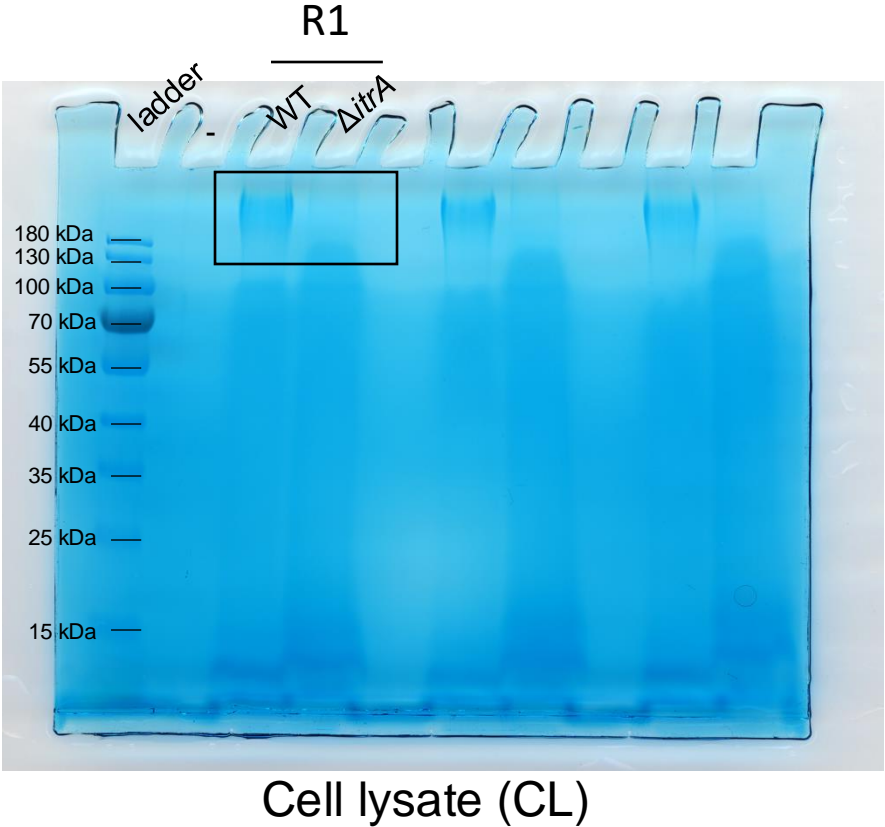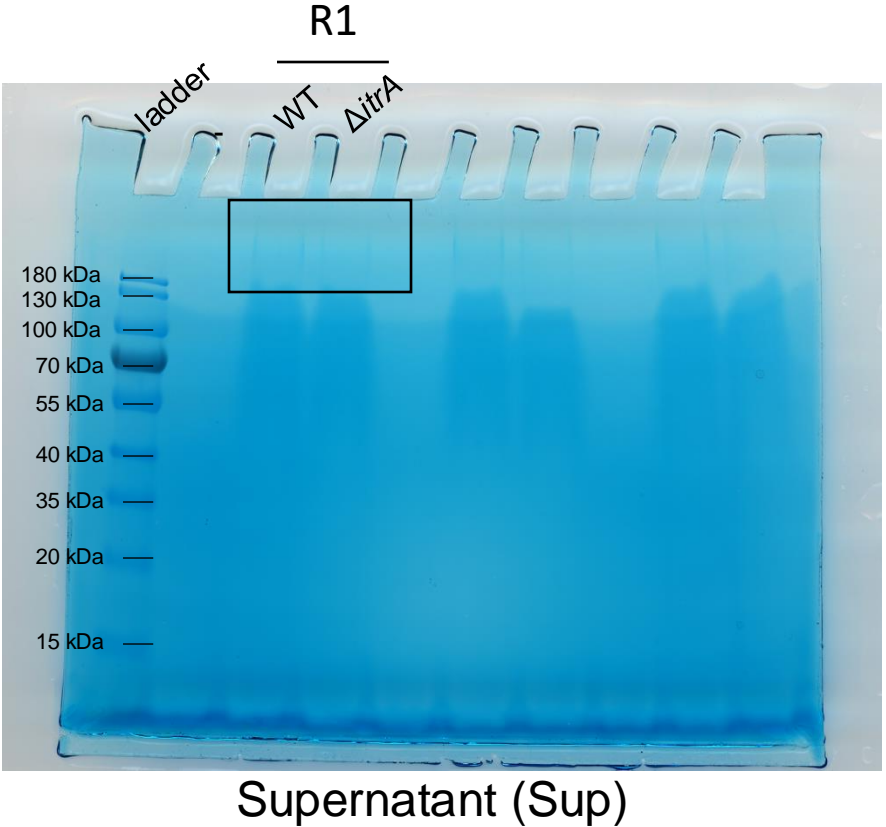

Supplement: Figure 1—source data 1. [file elife-101032-fig1-data1.pdf]

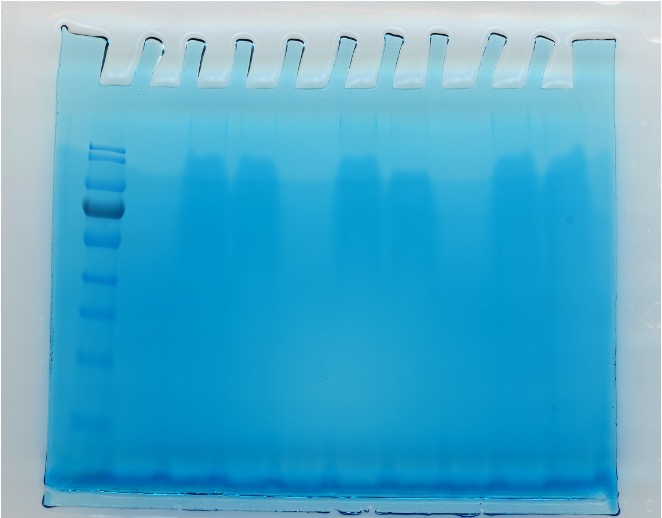

Supplement: Figure 1—source data 2. [file elife-101032-fig1-data2.zip › SUP.jpg]

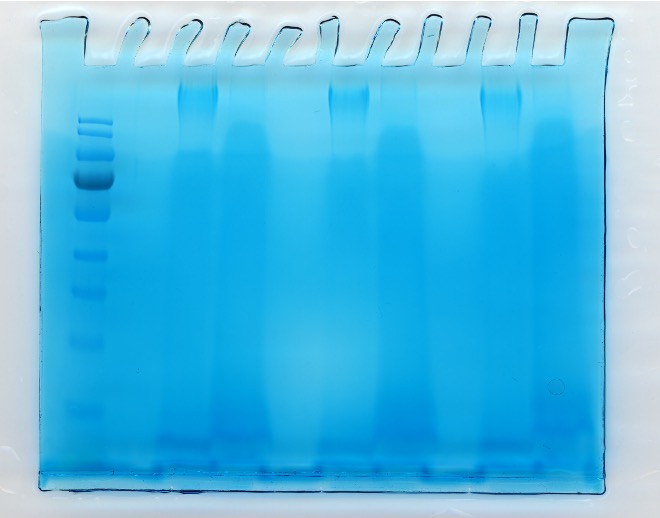

Supplement: Figure 1—source data 2. [file elife-101032-fig1-data2.zip › CL.jpg]

Figure 2B– source data-annotated

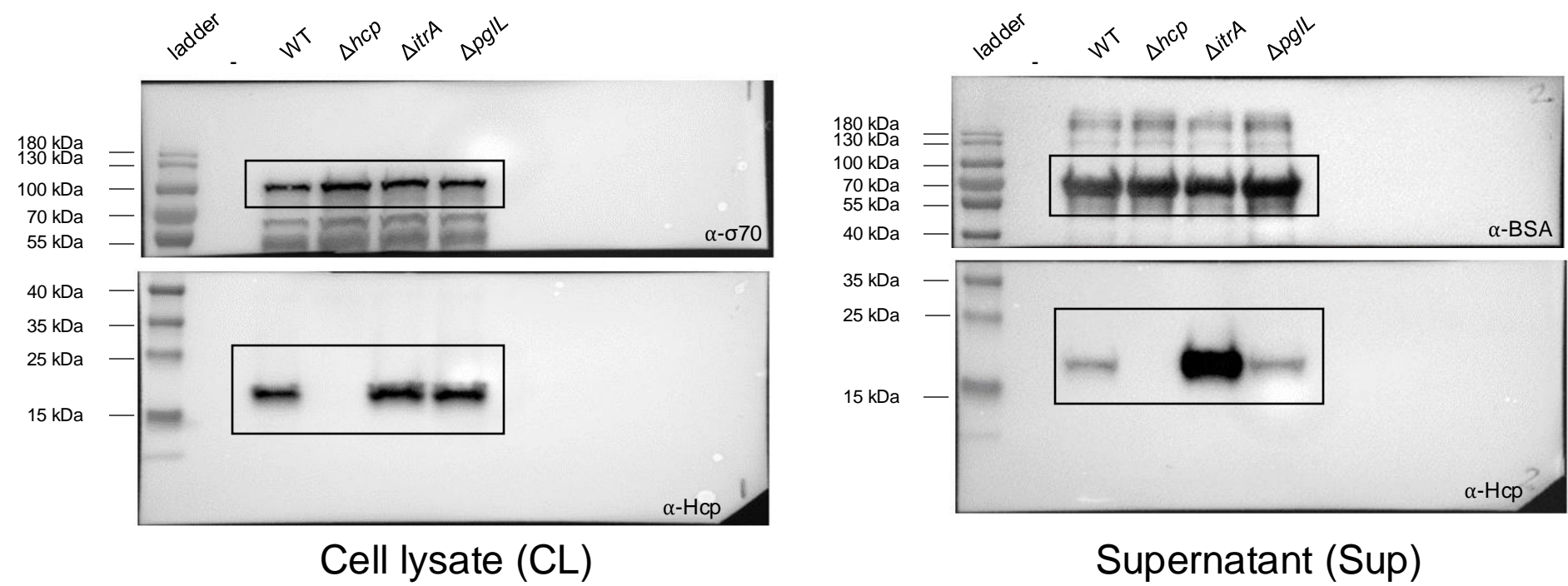

Supplement: Figure 2—source data 1. [file elife-101032-fig2-data1.pdf]

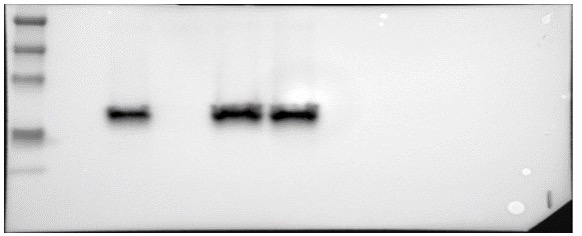

Supplement: Figure 2—source data 2. [file elife-101032-fig2-data2.zip › CL_Hcp.jpg]

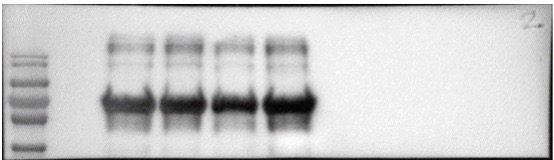

Supplement: Figure 2—source data 2. [file elife-101032-fig2-data2.zip › SUP_BSA.jpg]

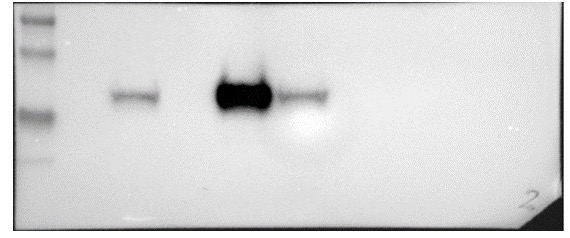

Supplement: Figure 2—source data 2. [file elife-101032-fig2-data2.zip › SUP_HcpA.jpg]

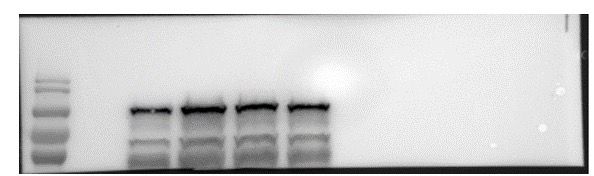

Supplement: Figure 2—source data 2. [file elife-101032-fig2-data2.zip › CL_Sigma70.jpg]

Figure 3B – source data-annotated

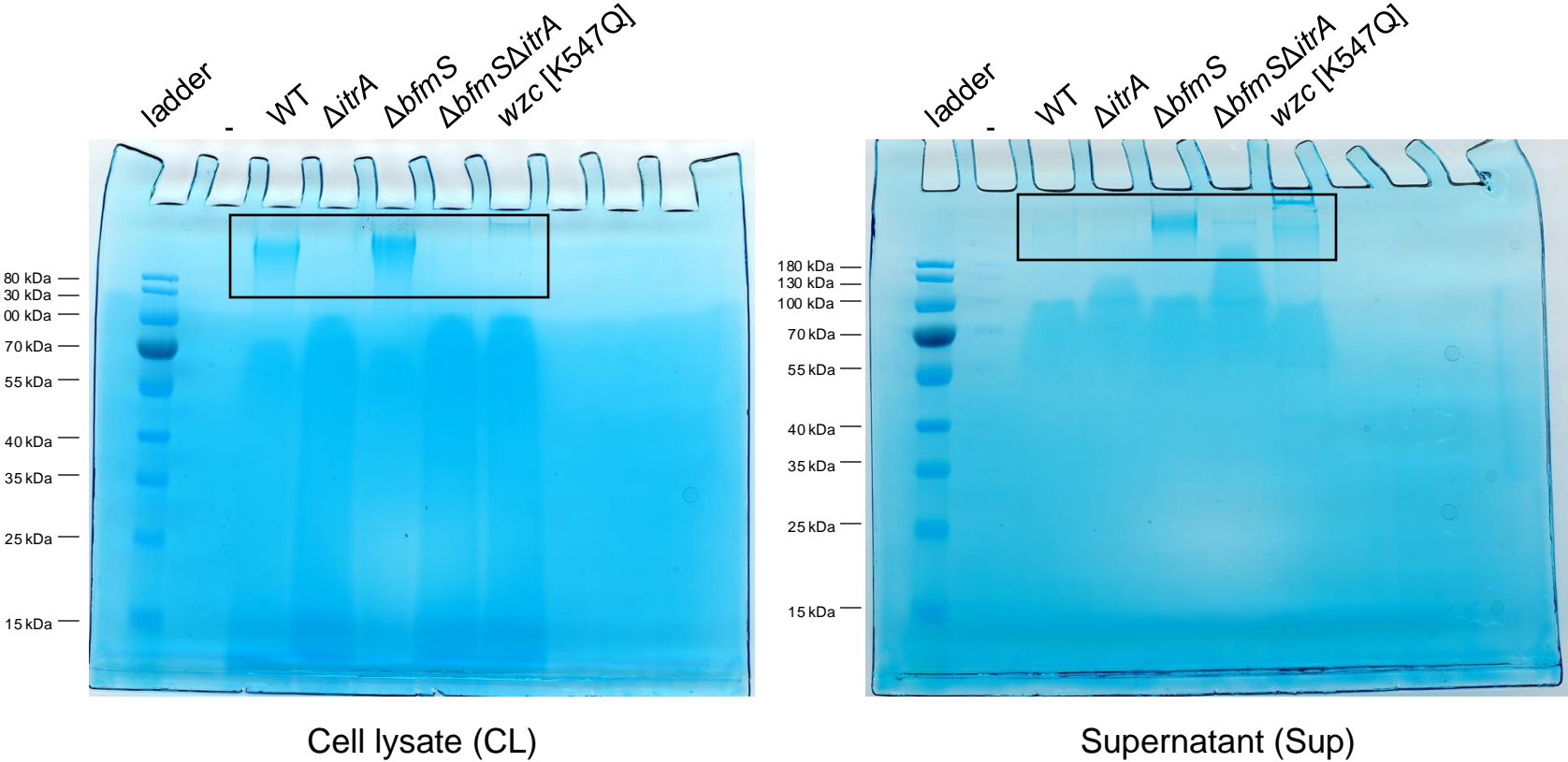

Supplement: Figure 3—source data 1. [file elife-101032-fig3-data1.pdf]

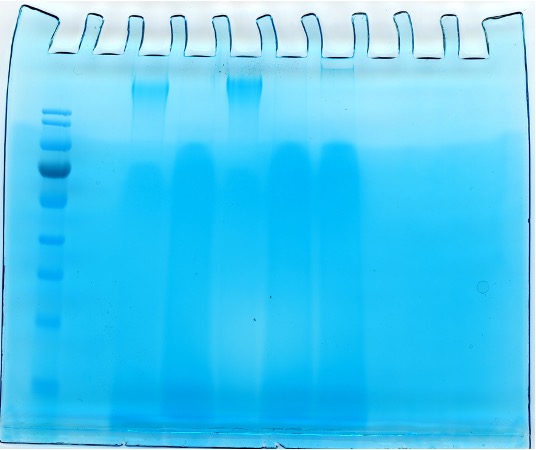

Supplement: Figure 3—source data 2. [file elife-101032-fig3-data2.zip › CL_Alcian.jpg]

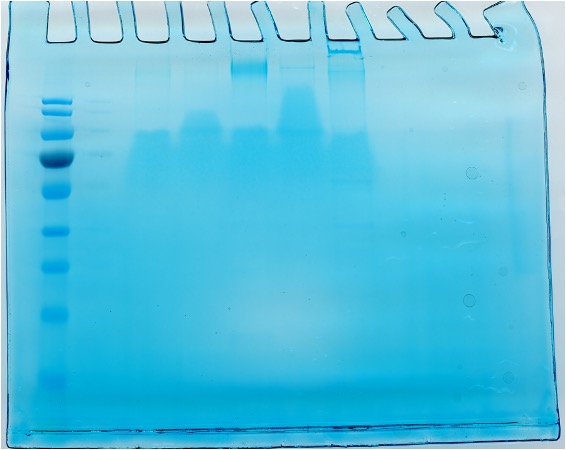

Supplement: Figure 3—source data 2. [file elife-101032-fig3-data2.zip › SUP_Alcian.jpg]

Figure 3D – source data-annotated

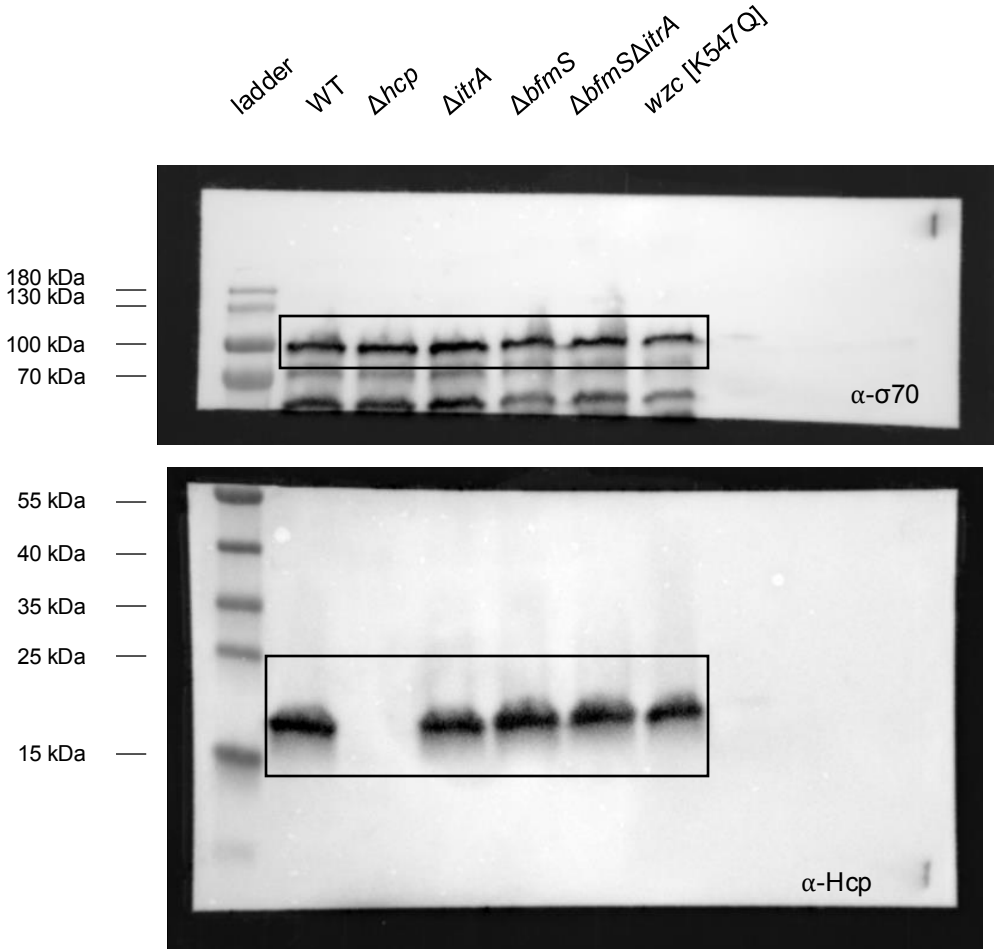

Cell lysate (CL)

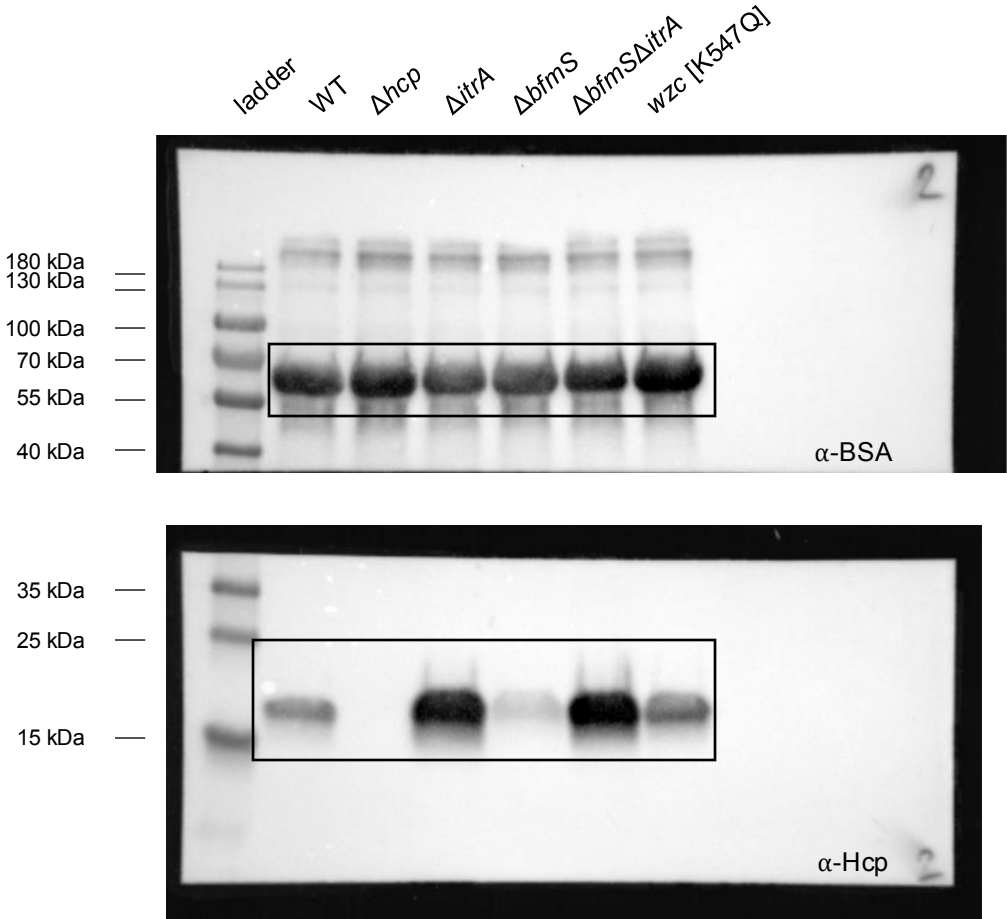

Supernatant (Sup)

Supplement: Figure 3—source data 3. [file elife-101032-fig3-data3.pdf]

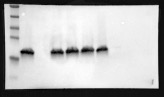

Supplement: Figure 3—source data 4. [file elife-101032-fig3-data4.zip › CL_Hcp.jpg]

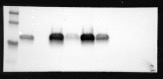

Supplement: Figure 3—source data 4. [file elife-101032-fig3-data4.zip › SUP_Hcp.jpg]

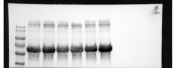

Supplement: Figure 3—source data 4. [file elife-101032-fig3-data4.zip › SUP_BSA.jpg]

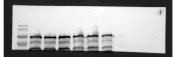

Supplement: Figure 3—source data 4. [file elife-101032-fig3-data4.zip › CL_Sigma70.jpg]

Figure 3-supplement 1D – source data-annotated

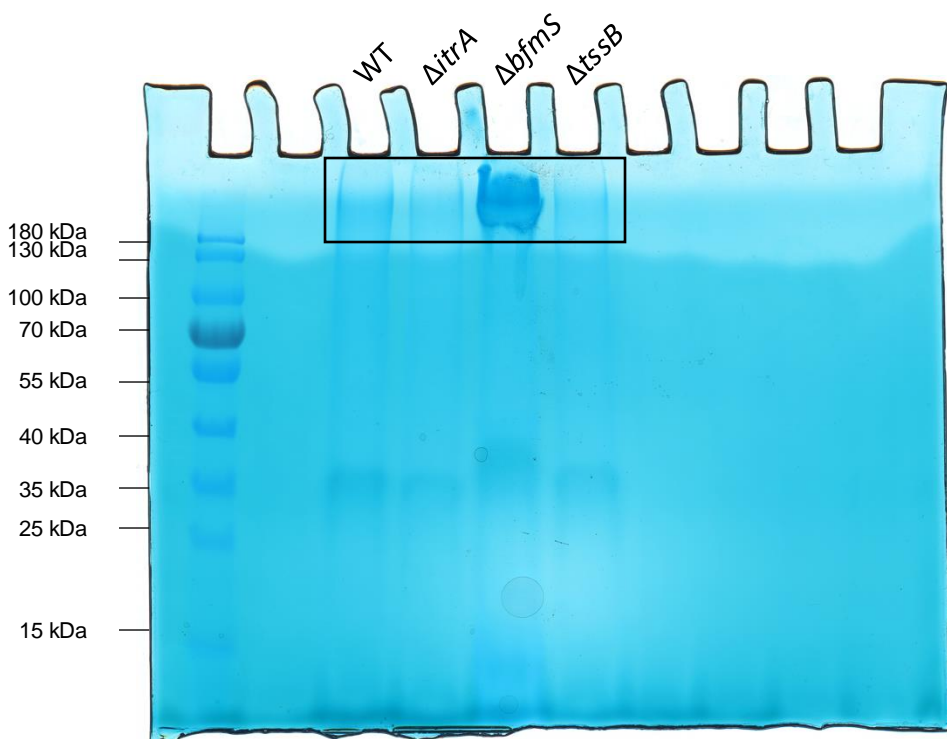

Supplement: Figure 3—figure supplement 1—source data 1. [file elife-101032-fig3-figsupp1-data1.pdf]

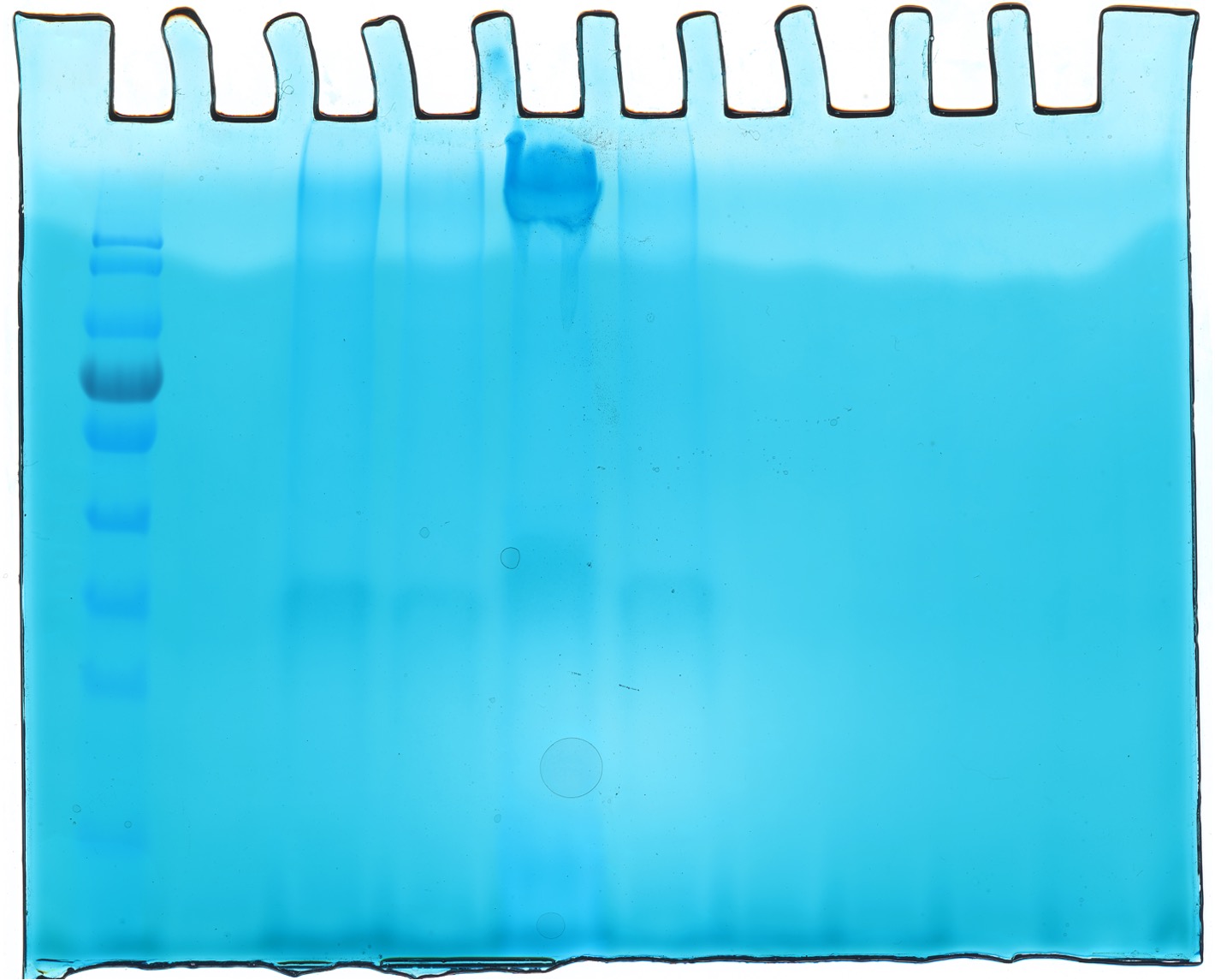

Supplement: Figure 3—figure supplement 1—source data 2. [file elife-101032-fig3-figsupp1-data2.zip › Gel.jpg]

Figure 3-supplement 1E – source data-annotated

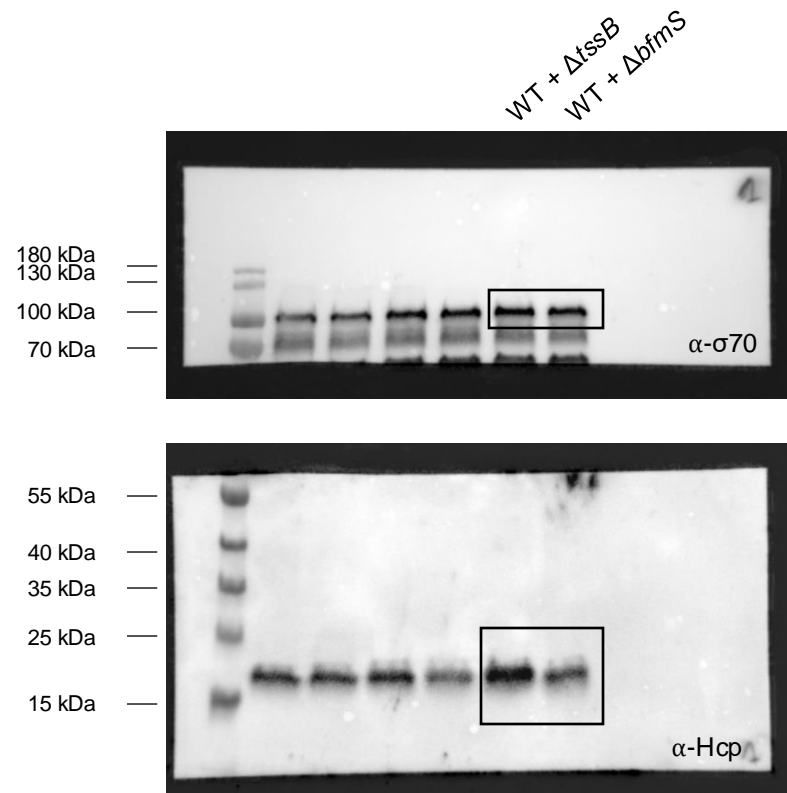

Cell lysate (CL)

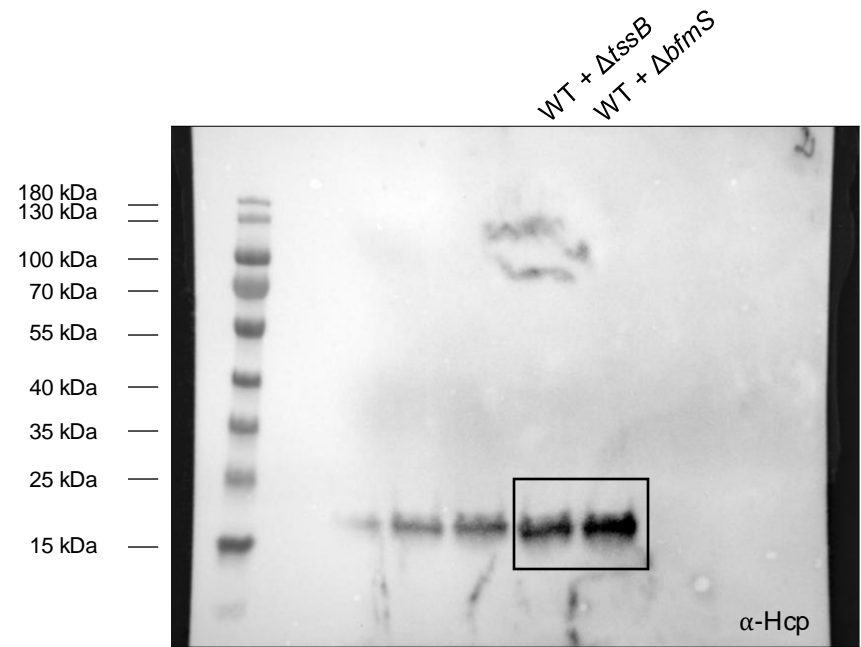

Supernatant (Sup)

Supplement: Figure 3—figure supplement 1—source data 3. [file elife-101032-fig3-figsupp1-data3.pdf]

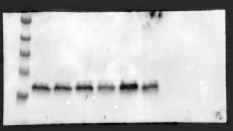

Supplement: Figure 3—figure supplement 1—source data 4. [file elife-101032-fig3-figsupp1-data4.zip › CL_Hcp.jpg]

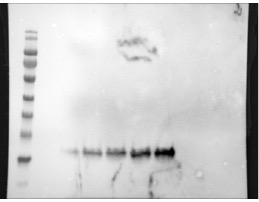

Supplement: Figure 3—figure supplement 1—source data 4. [file elife-101032-fig3-figsupp1-data4.zip › SUP_Hcp.jpg]

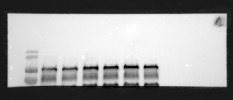

Supplement: Figure 3—figure supplement 1—source data 4. [file elife-101032-fig3-figsupp1-data4.zip › CL_Sigma70.jpg]

Figure 4A – source data-annotated

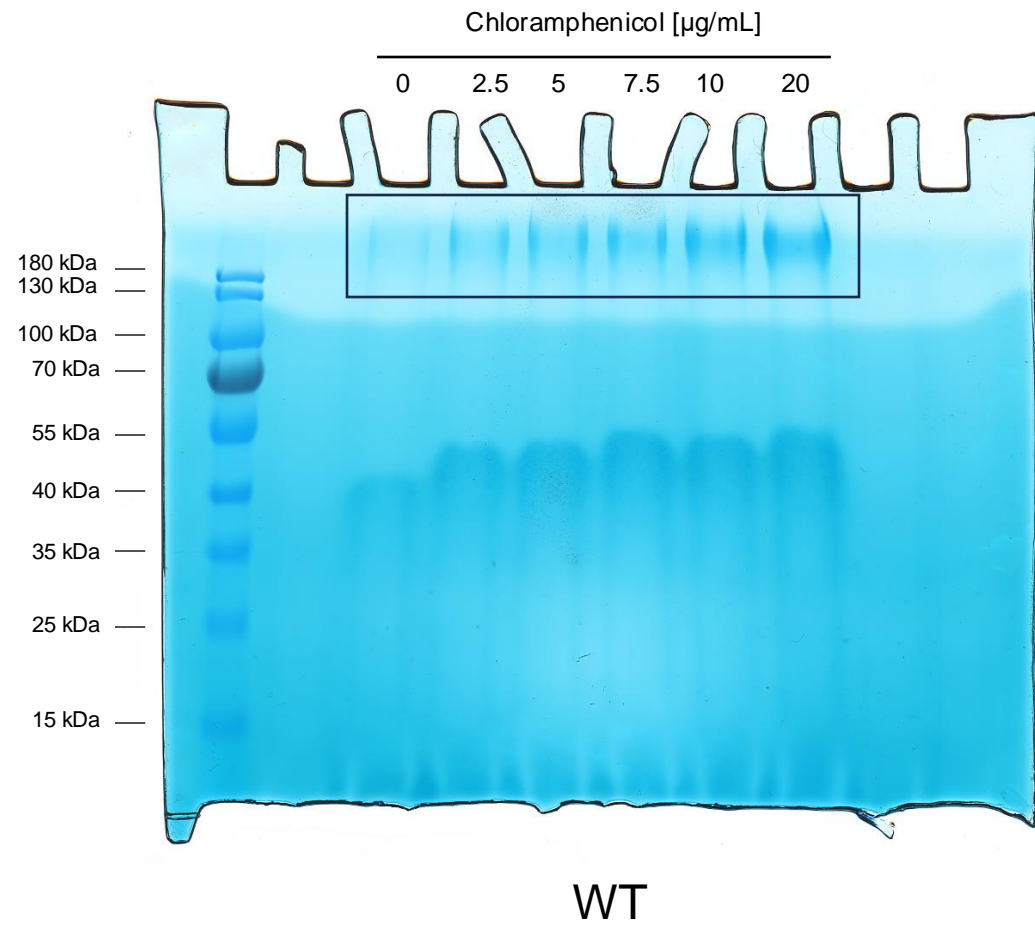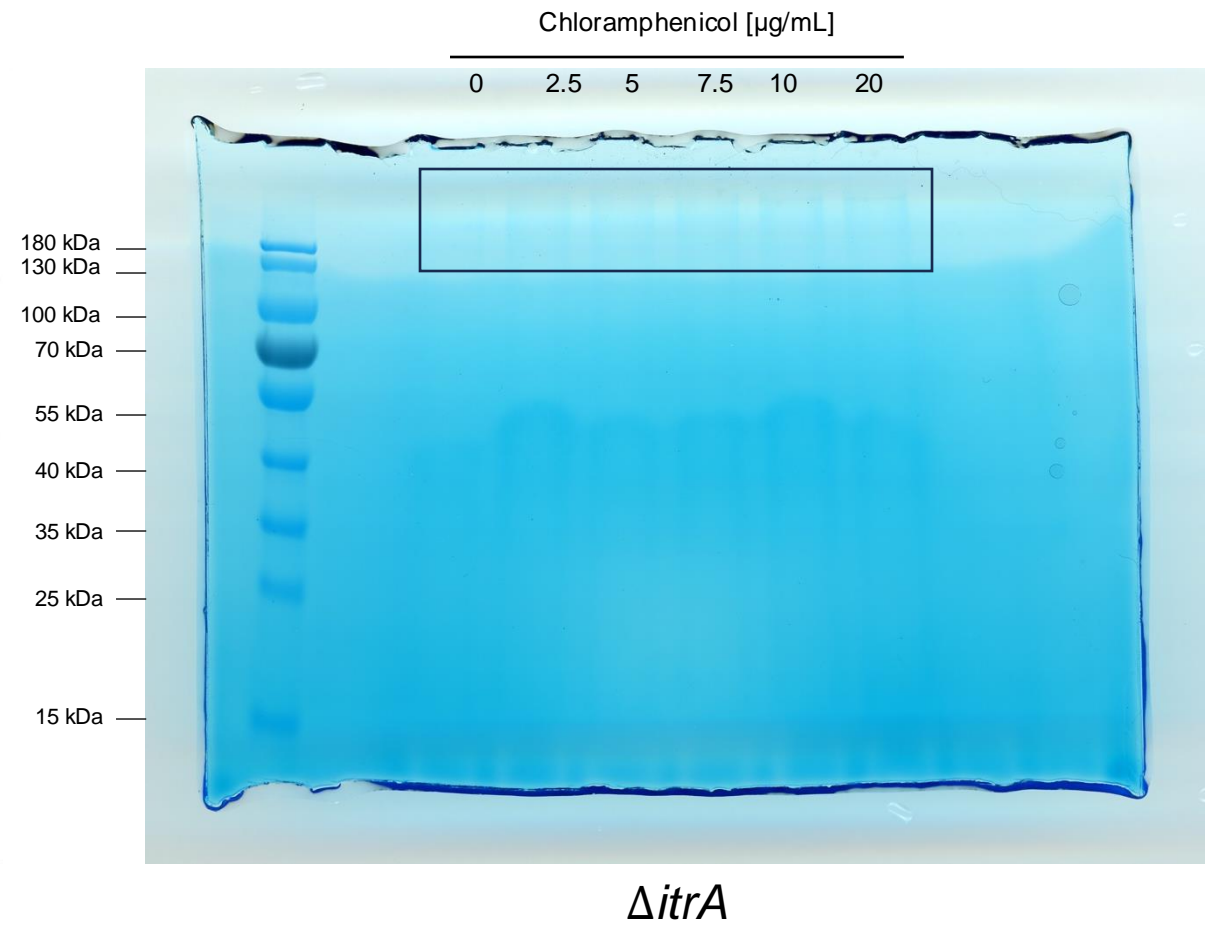

Supplement: Figure 4—source data 1. [file elife-101032-fig4-data1.pdf]

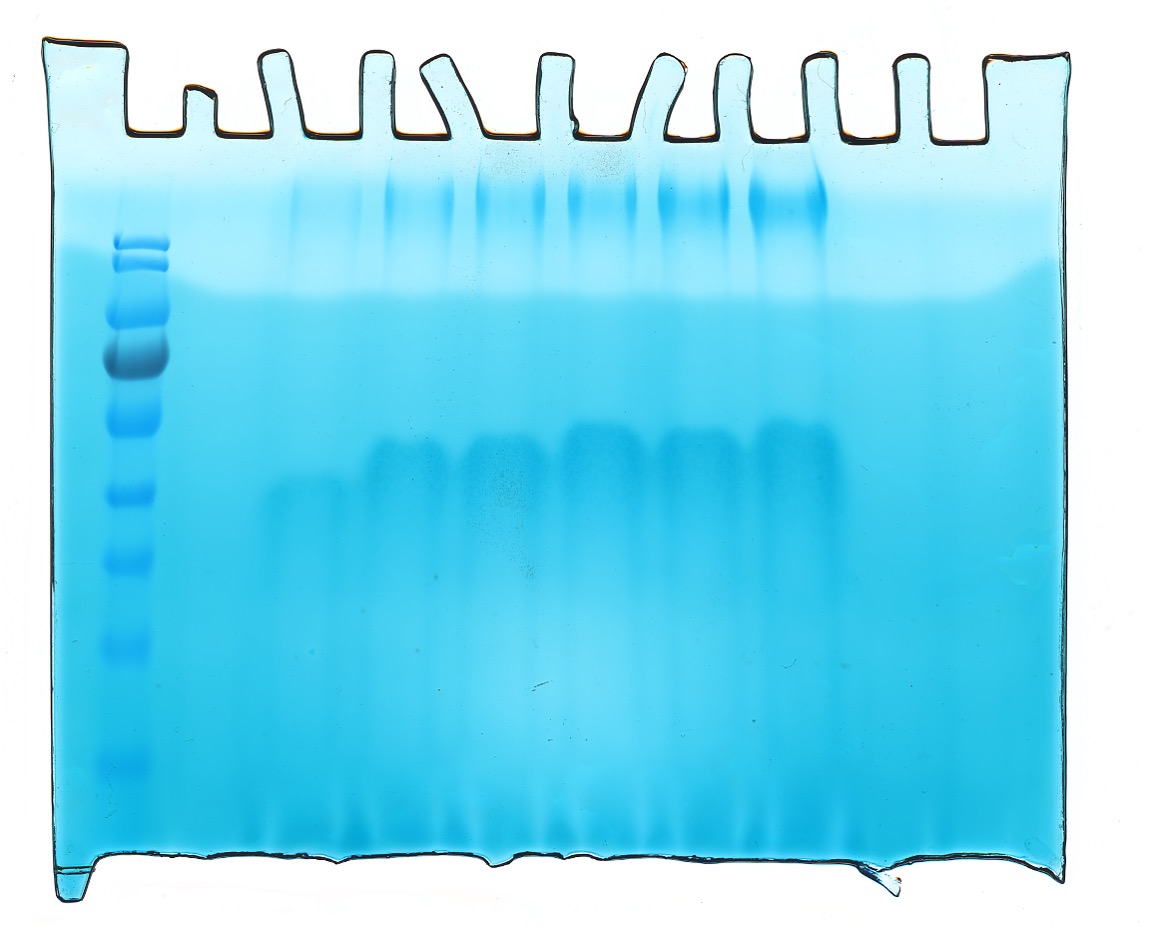

Supplement: Figure 4—source data 2. [file elife-101032-fig4-data2.zip › WT_chloro.jpg]

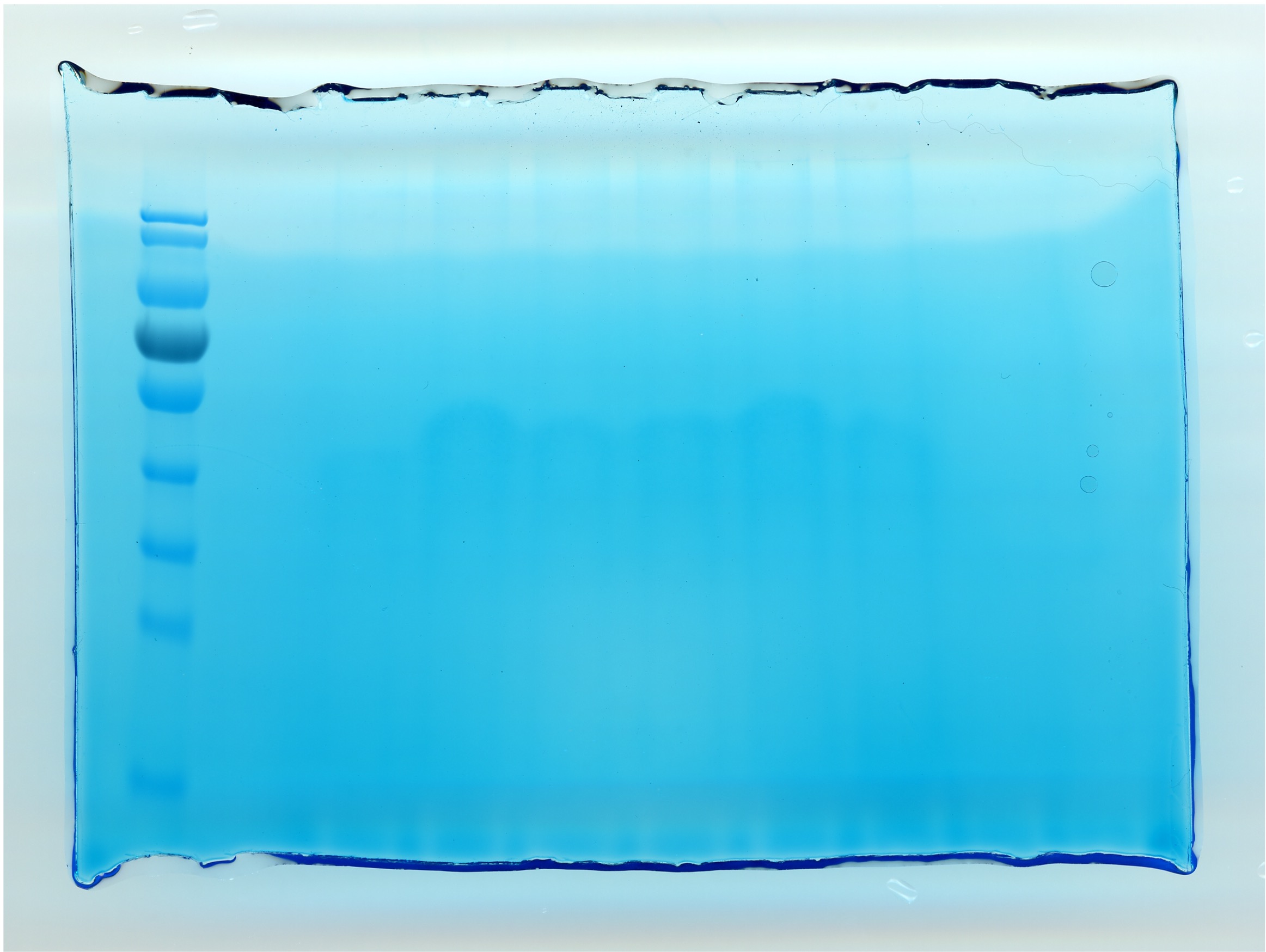

Supplement: Figure 4—source data 2. [file elife-101032-fig4-data2.zip › itrA_chloro.jpg]

Figure 4B– source data-annotated

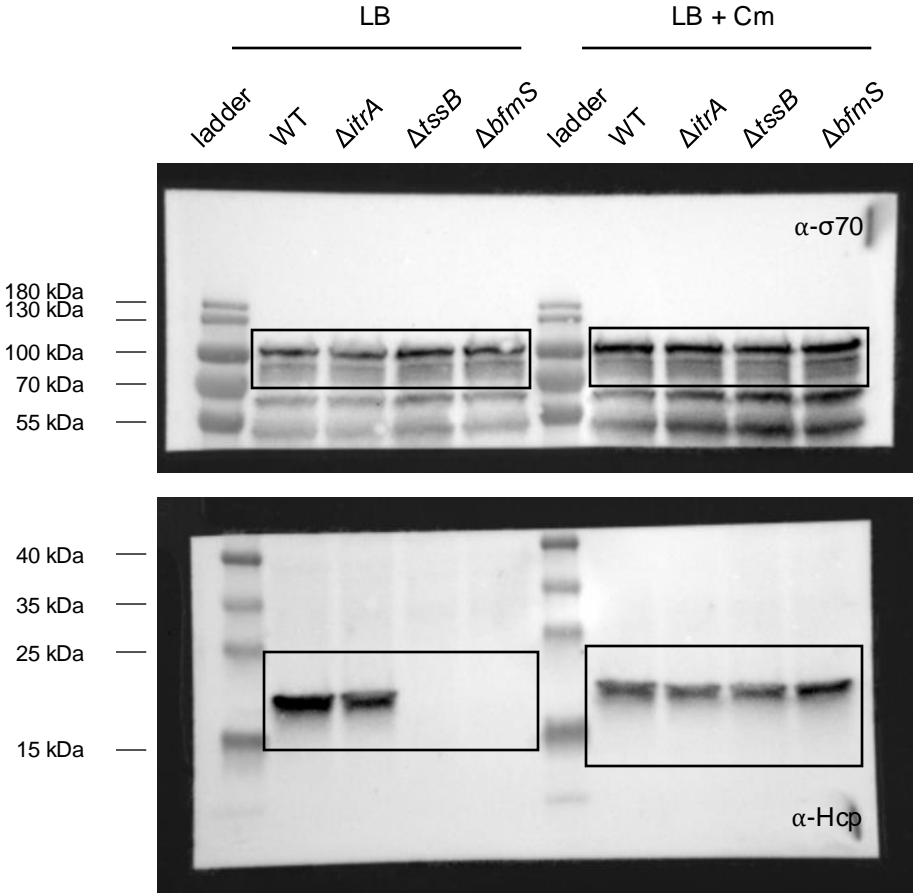

Cell lysate (CL)

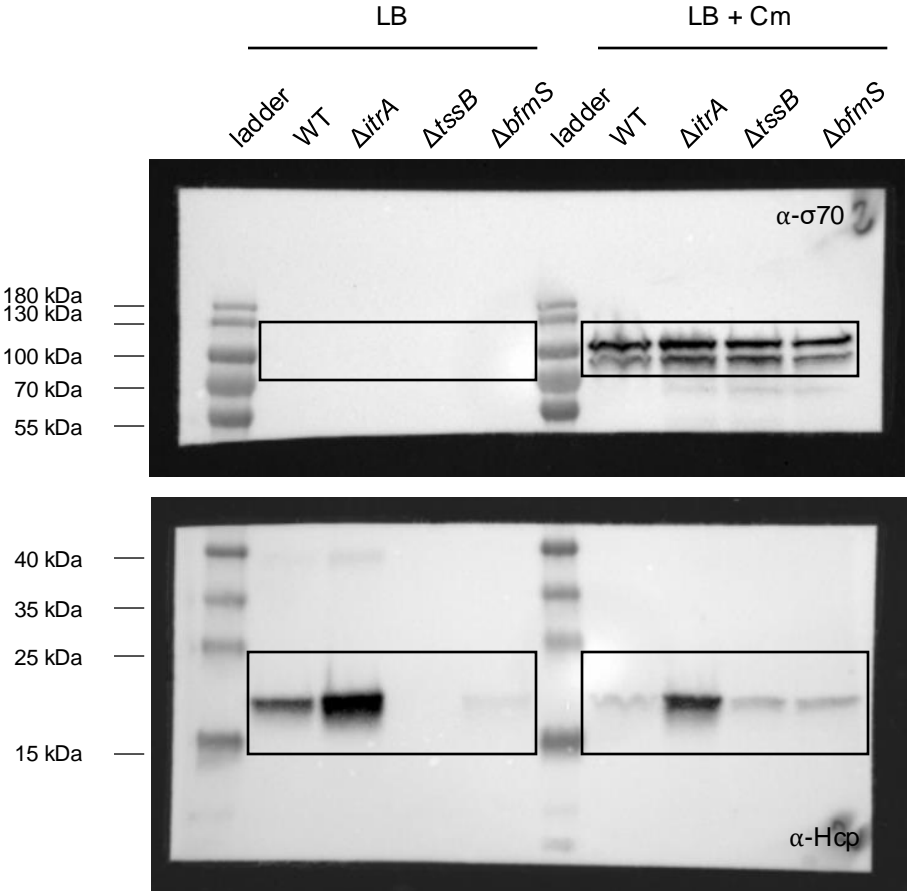

Supernatant (Sup)

Supplement: Figure 4—source data 3. [file elife-101032-fig4-data3.pdf]

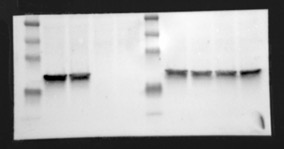

Supplement: Figure 4—source data 4. [file elife-101032-fig4-data4.zip › CL_Hcp.jpg]

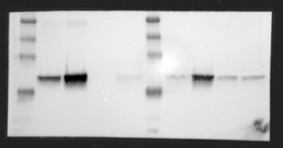

Supplement: Figure 4—source data 4. [file elife-101032-fig4-data4.zip › SUP_Hcp.jpg]

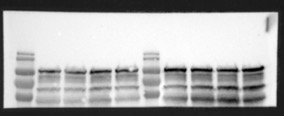

Supplement: Figure 4—source data 4. [file elife-101032-fig4-data4.zip › CL_Sigma70.jpg]

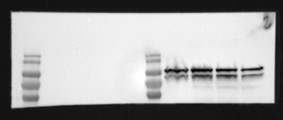

Supplement: Figure 4—source data 4. [file elife-101032-fig4-data4.zip › SUP_Sigma70.jpg]

Figure 5D – source data-annotated

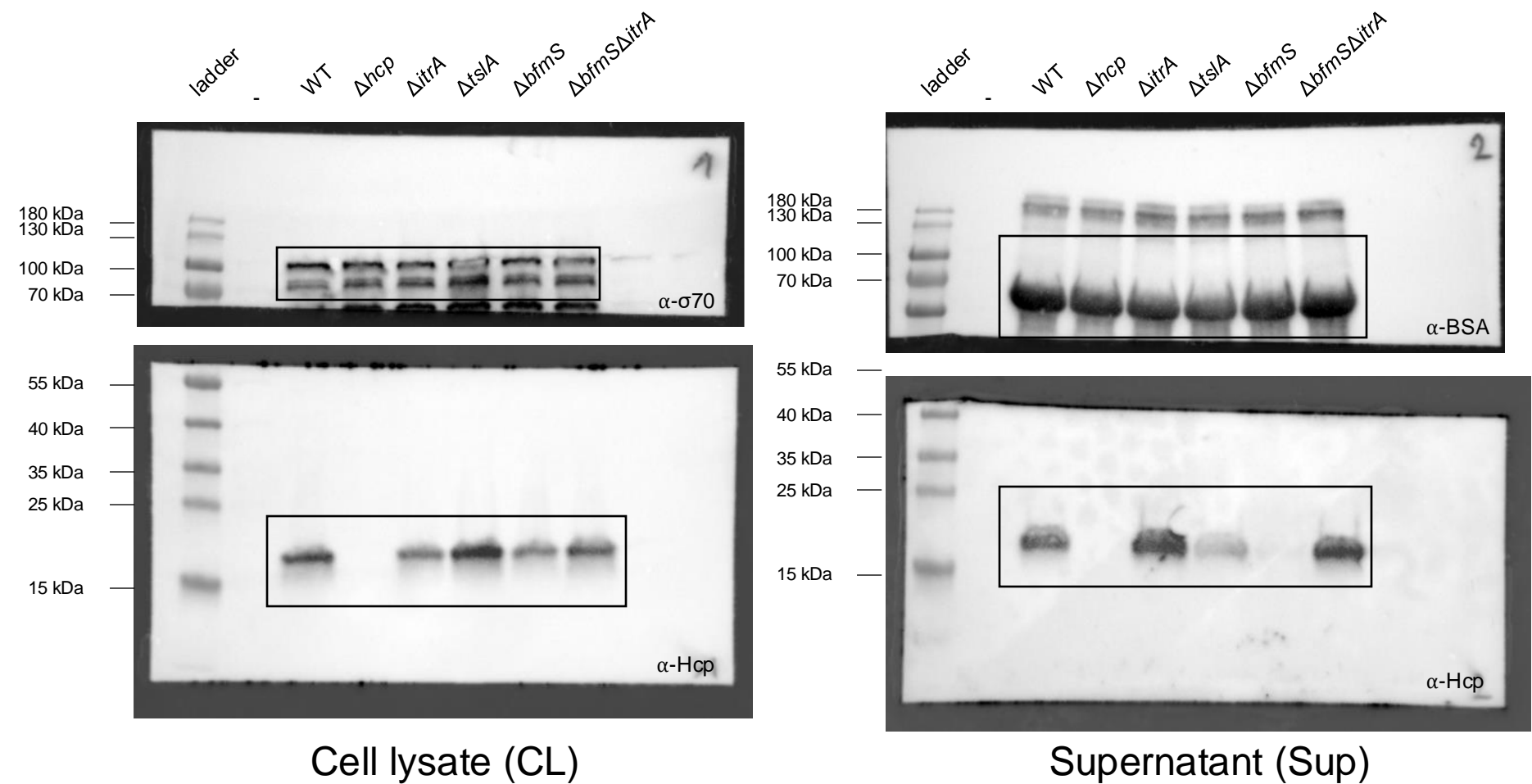

Supplement: Figure 5—source data 1. [file elife-101032-fig5-data1.pdf]

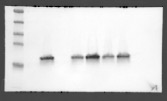

Supplement: Figure 5—source data 2. [file elife-101032-fig5-data2.zip › CL_Hcp.jpg]

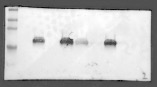

Supplement: Figure 5—source data 2. [file elife-101032-fig5-data2.zip › SUP_Hcp.jpg]

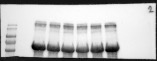

Supplement: Figure 5—source data 2. [file elife-101032-fig5-data2.zip › SUP_BSA.jpg]

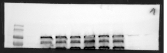

Supplement: Figure 5—source data 2. [file elife-101032-fig5-data2.zip › CL_Sigma70.png]

Figure 5-supplement 1B – source data-annotated

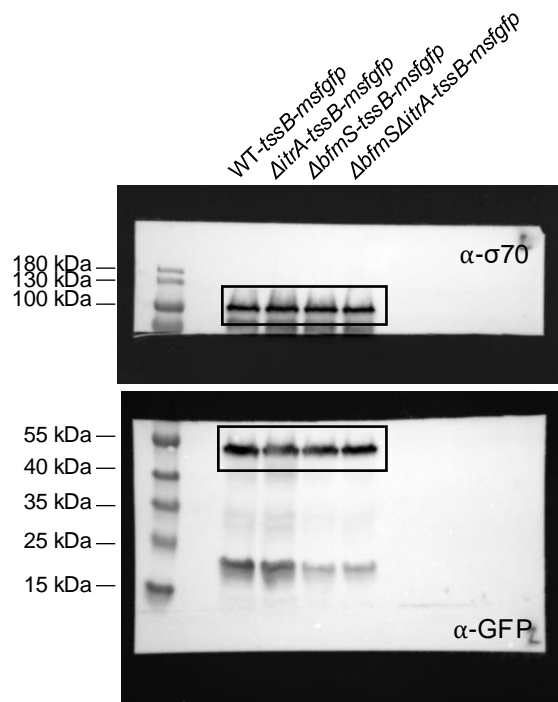

Supplement: Figure 5—figure supplement 1—source data 1. [file elife-101032-fig5-figsupp1-data1.pdf]

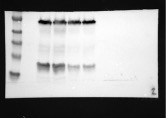

Supplement: Figure 5—figure supplement 1—source data 2. [file elife-101032-fig5-figsupp1-data2.zip › GFP.jpg]

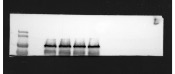

Supplement: Figure 5—figure supplement 1—source data 2. [file elife-101032-fig5-figsupp1-data2.zip › Sigma70.jpg]

Figure 6A– source data

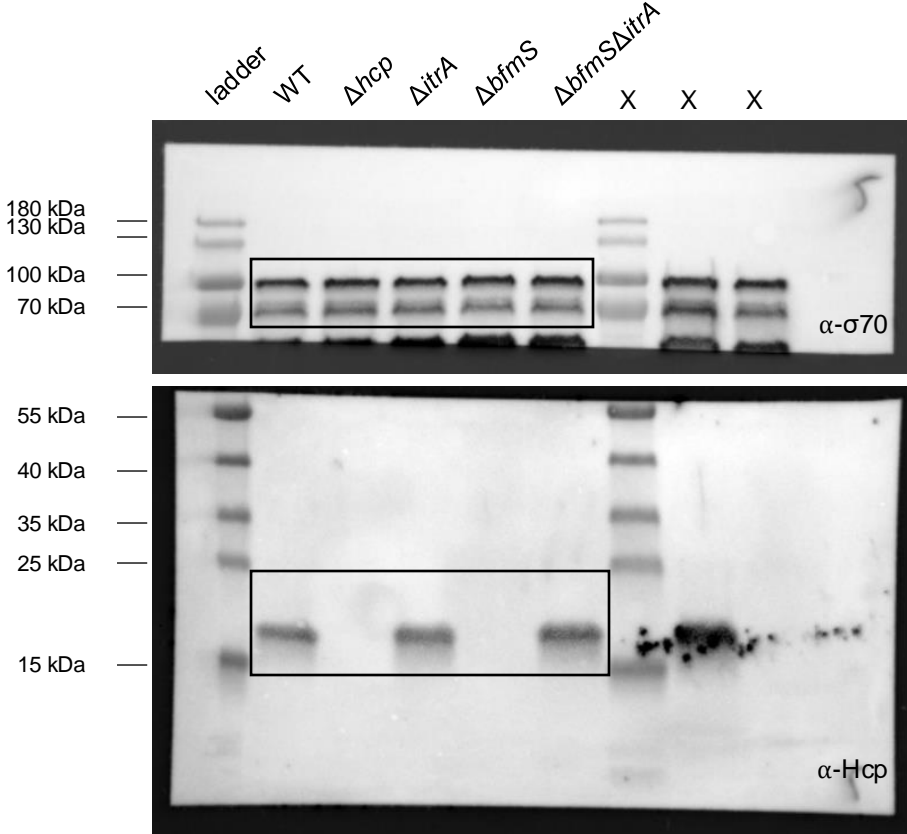

Supplement: Figure 6—source data 1. [file elife-101032-fig6-data1.pdf]

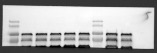

Supplement: Figure 6—source data 2. [file elife-101032-fig6-data2.zip › Sigma70.jpg]

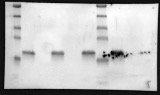

Supplement: Figure 6—source data 2. [file elife-101032-fig6-data2.zip › Hcp.jpg]

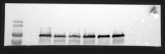

Supplement: Figure 6—source data 4. [file elife-101032-fig6-data4.zip › STAT_CL_Sigma70.jpg]

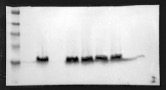

Supplement: Figure 6—source data 4. [file elife-101032-fig6-data4.zip › EXP_CL_Hcp.jpg]

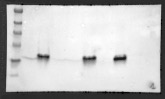

Supplement: Figure 6—source data 4. [file elife-101032-fig6-data4.zip › STAT_CL_Hcp.jpg]

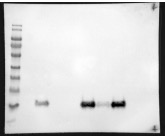

Supplement: Figure 6—source data 4. [file elife-101032-fig6-data4.zip › EXP_SUP_Hcp.jpg]

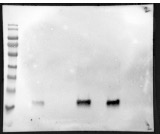

Supplement: Figure 6—source data 4. [file elife-101032-fig6-data4.zip › STAT_SUP_Hcp.jpg]

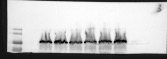

Supplement: Figure 6—source data 4. [file elife-101032-fig6-data4.zip › EXP_CL_Sigma70.jpg]

Figure 6C – source data-annotated

+ = WT  
 $\Delta$  =  $\Delta$ tssB

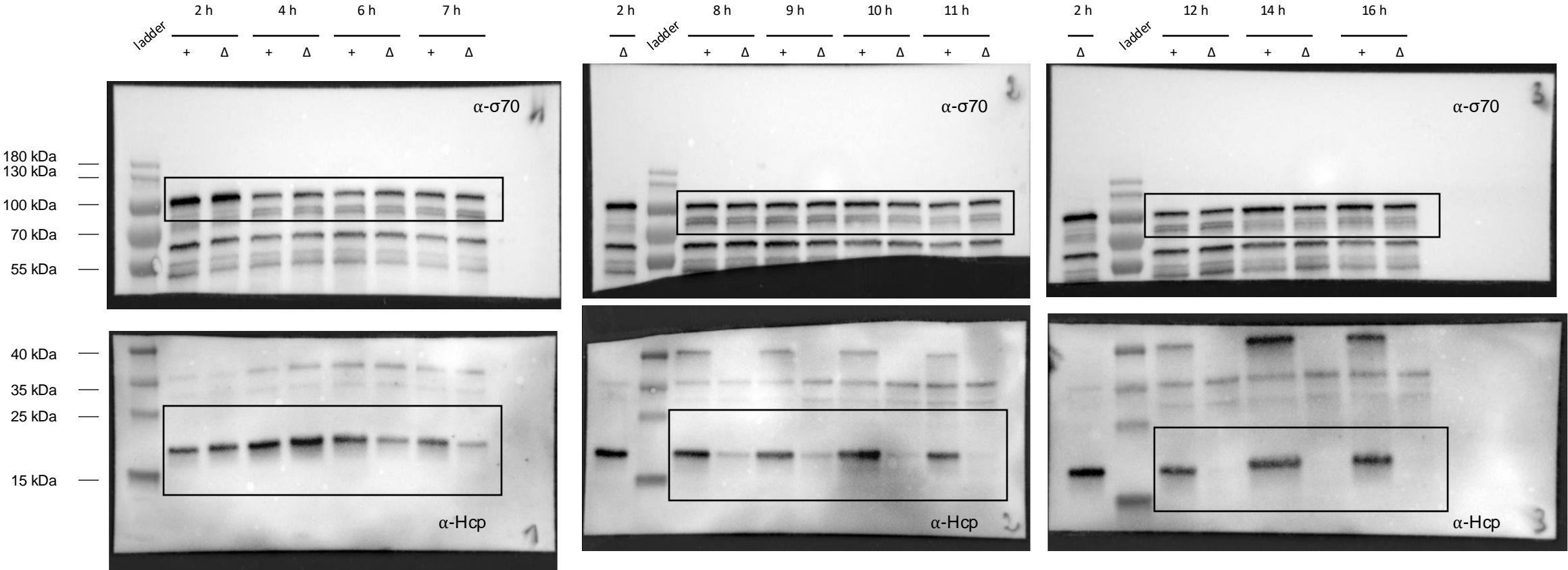

Supplement: Figure 6—source data 5. [file elife-101032-fig6-data5.pdf]

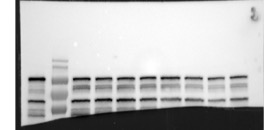

Supplement: Figure 6—source data 6. [file elife-101032-fig6-data6.zip › 8-11h_Sigma70.jpg]

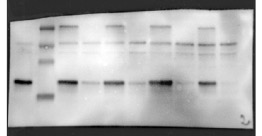

Supplement: Figure 6—source data 6. [file elife-101032-fig6-data6.zip › 8-11h_Hcp.jpg]

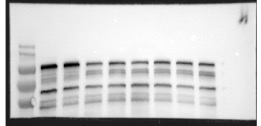

Supplement: Figure 6—source data 6. [file elife-101032-fig6-data6.zip › 2-7h_Sigma70.jpg]

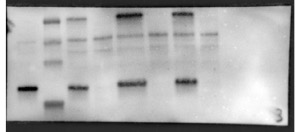

Supplement: Figure 6—source data 6. [file elife-101032-fig6-data6.zip › 12-16h_Hcp.jpg]

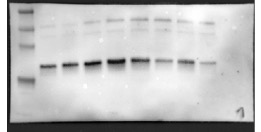

Supplement: Figure 6—source data 6. [file elife-101032-fig6-data6.zip › 2-7h_Hcp.jpg]

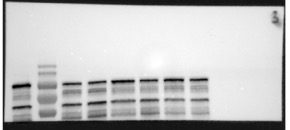

Supplement: Figure 6—source data 6. [file elife-101032-fig6-data6.zip › 12-16h_Sigma70.jpg]

Figure 6-supplement 1A – source data

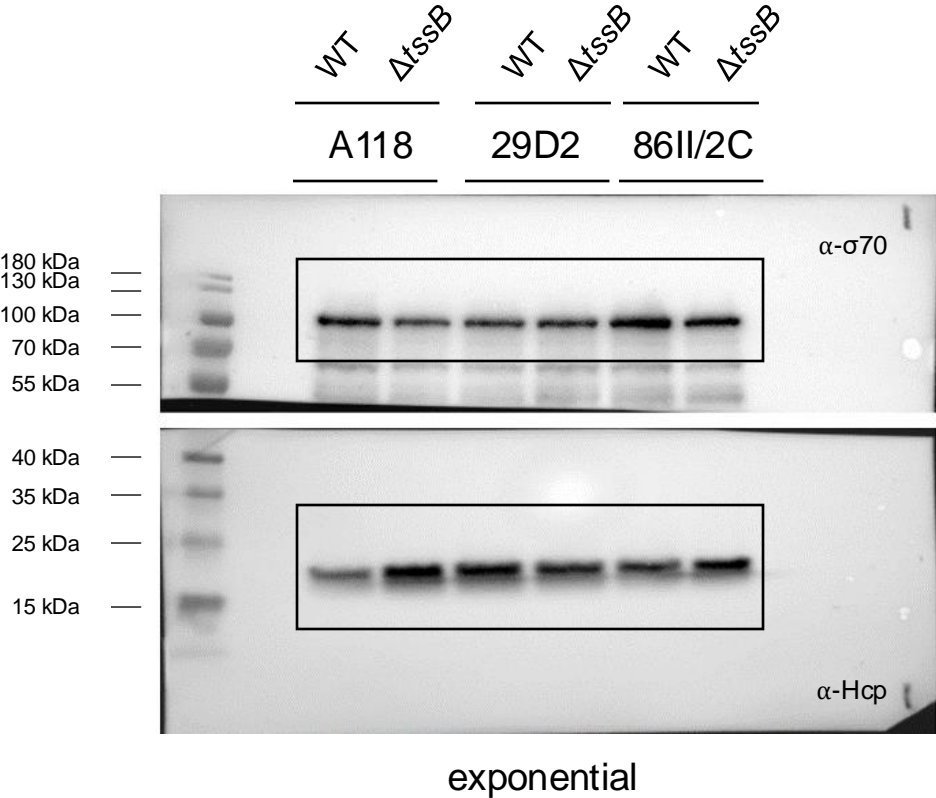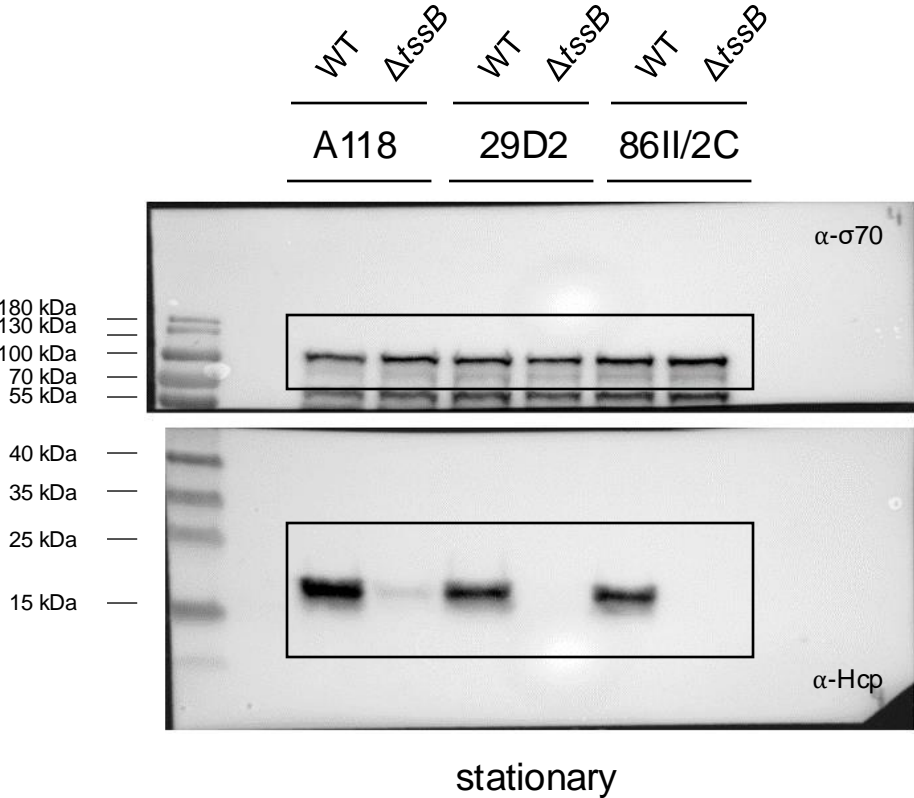

Supplement: Figure 6—figure supplement 1—source data 1. [file elife-101032-fig6-figsupp1-data1.pdf]

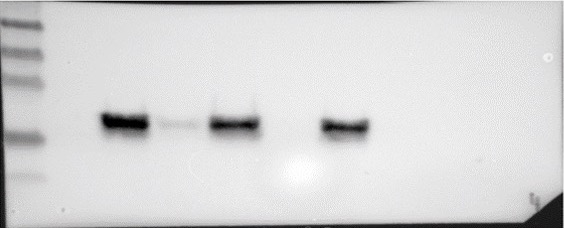

Supplement: Figure 6—figure supplement 1—source data 2. [file elife-101032-fig6-figsupp1-data2.zip › STAT_Hcp.jpg]

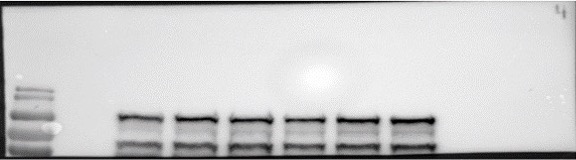

Supplement: Figure 6—figure supplement 1—source data 2. [file elife-101032-fig6-figsupp1-data2.zip › STAT_Sigma70.jpg]

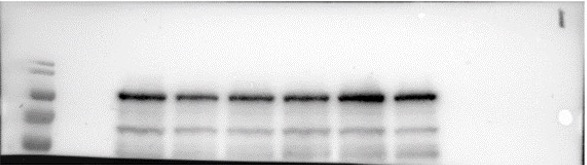

Supplement: Figure 6—figure supplement 1—source data 2. [file elife-101032-fig6-figsupp1-data2.zip › EXP_Sigma70.jpg]

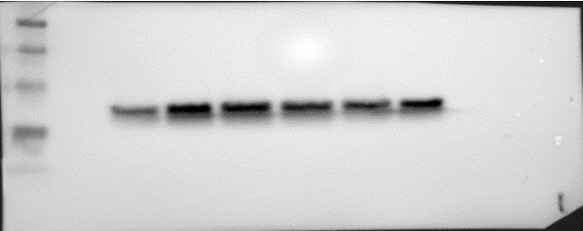

Supplement: Figure 6—figure supplement 1—source data 2. [file elife-101032-fig6-figsupp1-data2.zip › EXP_Hcp.jpg]

Figure 6-supplement 1B– source data-annotated

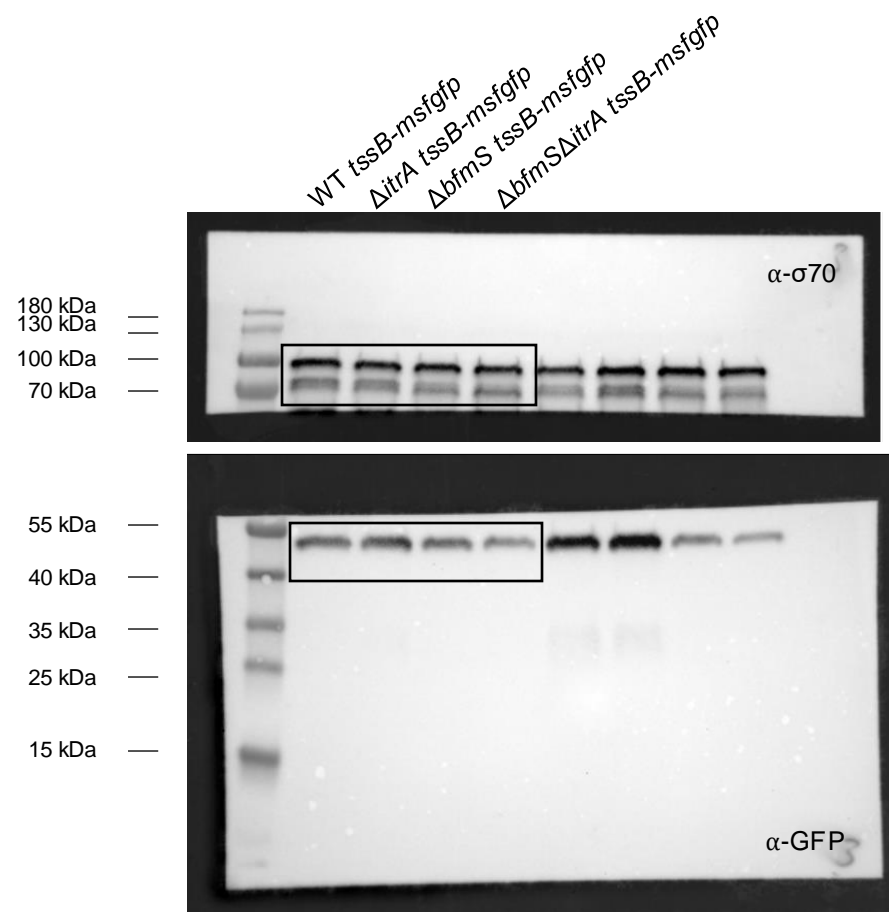

Supplement: Figure 6—figure supplement 1—source data 3. [file elife-101032-fig6-figsupp1-data3.pdf]

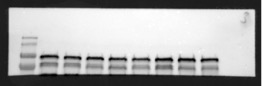

Supplement: Figure 6—figure supplement 1—source data 4. [file elife-101032-fig6-figsupp1-data4.zip › Sigma70.jpg]

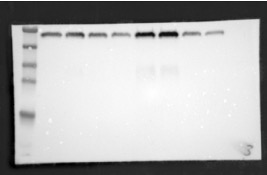

Supplement: Figure 6—figure supplement 1—source data 4. [file elife-101032-fig6-figsupp1-data4.zip › GFP.jpg]

Figure 7B – source data-annotated

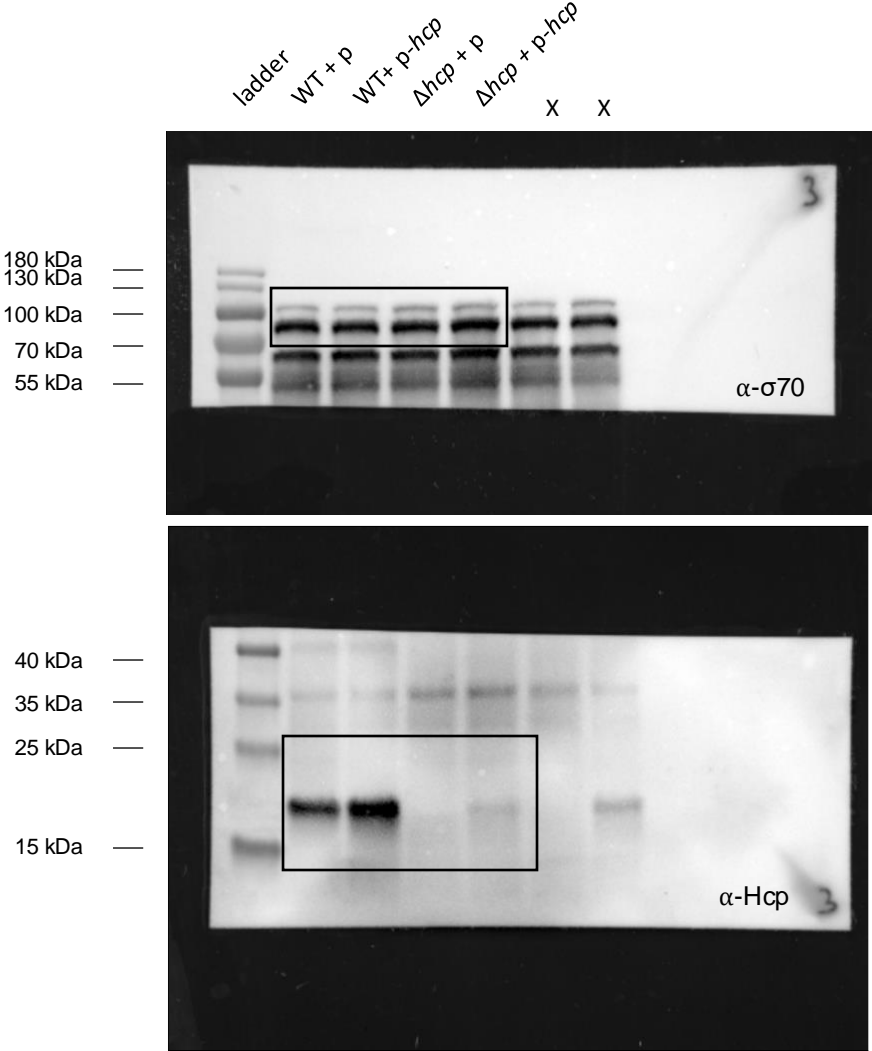

Supplement: Figure 7—source data 1. [file elife-101032-fig7-data1.pdf]

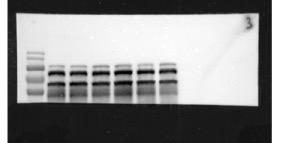

Supplement: Figure 7—source data 2. [file elife-101032-fig7-data2.zip › Sigma70.jpg]

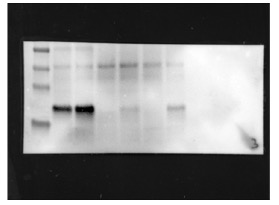

Supplement: Figure 7—source data 2. [file elife-101032-fig7-data2.zip › Hcp.jpg]

Figure 7C– source data-annotated

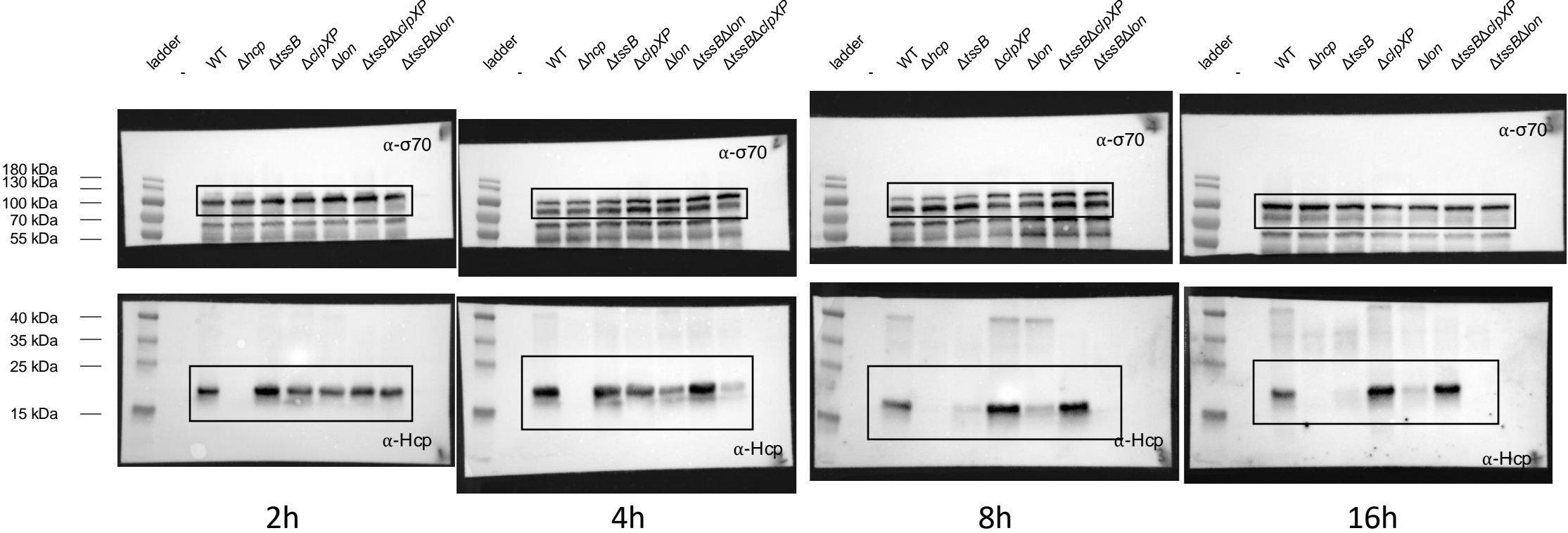

Supplement: Figure 7—source data 3. [file elife-101032-fig7-data3.pdf]

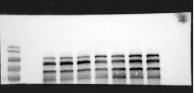

Supplement: Figure 7—source data 4. [file elife-101032-fig7-data4.zip › 8h_Sigma70.jpg]

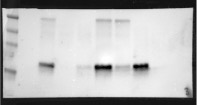

Supplement: Figure 7—source data 4. [file elife-101032-fig7-data4.zip › 8h_Hcp.jpg]

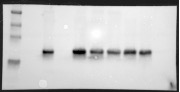

Supplement: Figure 7—source data 4. [file elife-101032-fig7-data4.zip › 2h_Hcp.jpg]

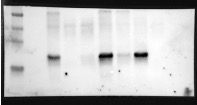

Supplement: Figure 7—source data 4. [file elife-101032-fig7-data4.zip › 16h_Hcp.jpg]

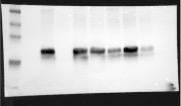

Supplement: Figure 7—source data 4. [file elife-101032-fig7-data4.zip › 4h_Hcp.jpg]

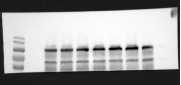

Supplement: Figure 7—source data 4. [file elife-101032-fig7-data4.zip › 2h_Sigma70.jpg]

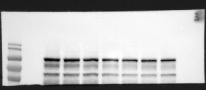

Supplement: Figure 7—source data 4. [file elife-101032-fig7-data4.zip › 16h_Sigma70.jpg]

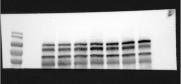

Supplement: Figure 7—source data 4. [file elife-101032-fig7-data4.zip › 4h_Sigma70.jpg]

Figure 7D – source data

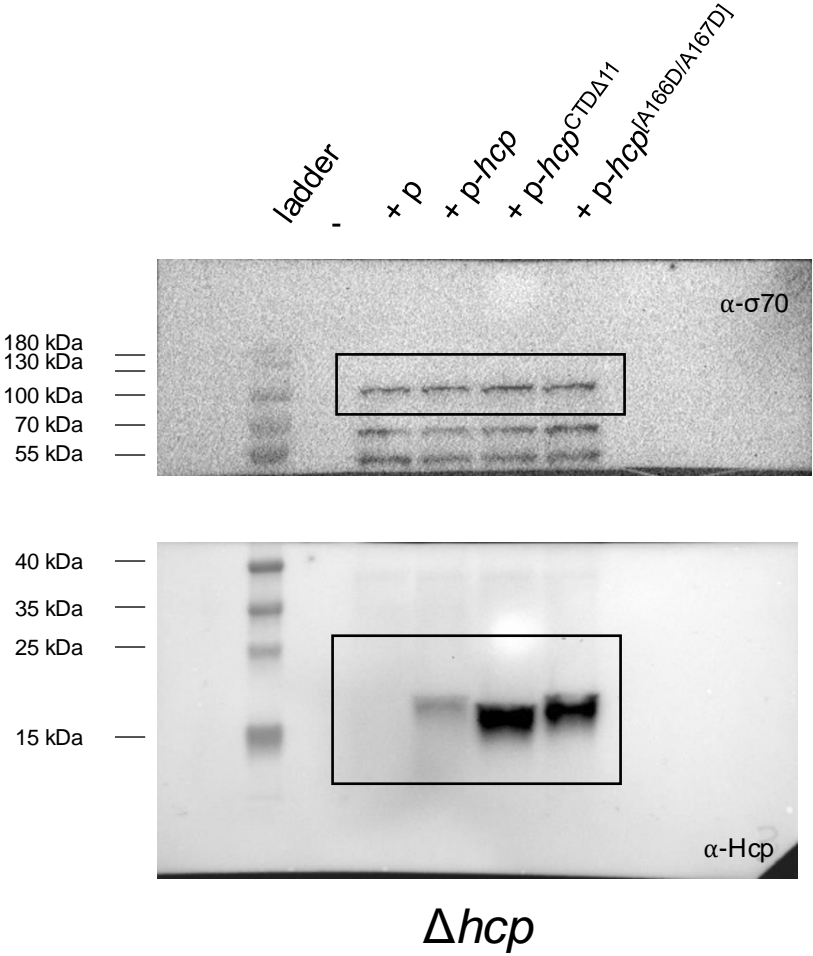

Supplement: Figure 7—source data 5. [file elife-101032-fig7-data5.pdf]

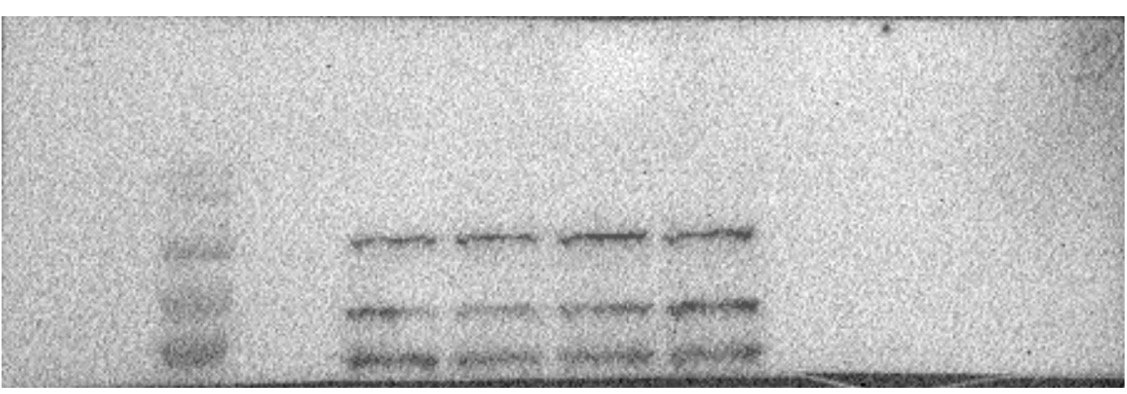

Supplement: Figure 7—source data 6. [file elife-101032-fig7-data6.zip › Sigma70.jpg]

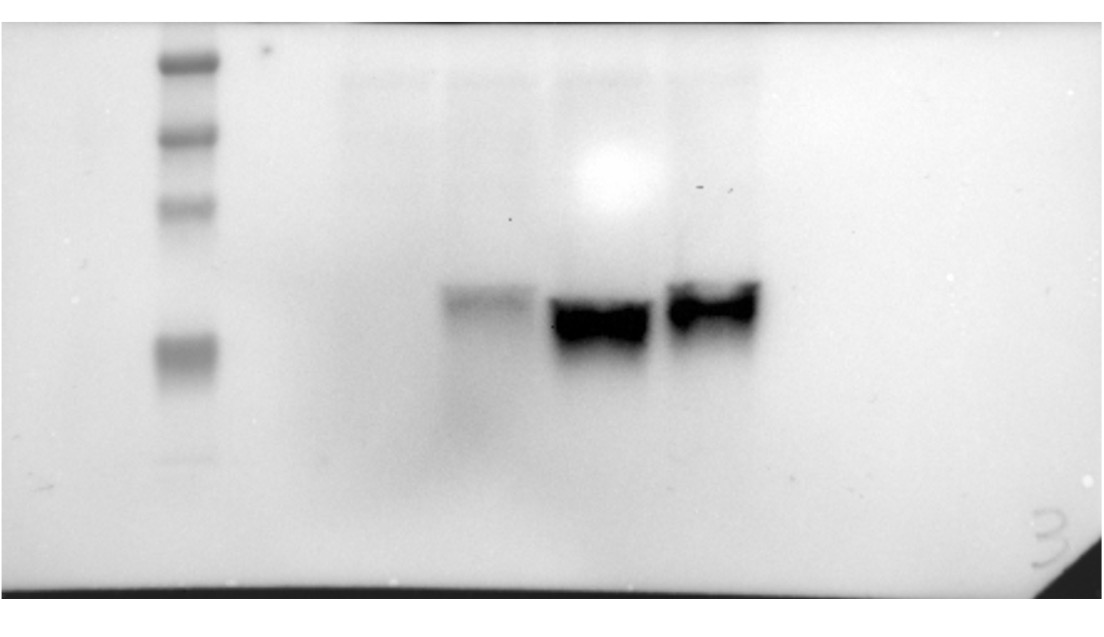

Supplement: Figure 7—source data 6. [file elife-101032-fig7-data6.zip › Hcp.jpg]

Figure 7-supplement 1A– source data-annotated

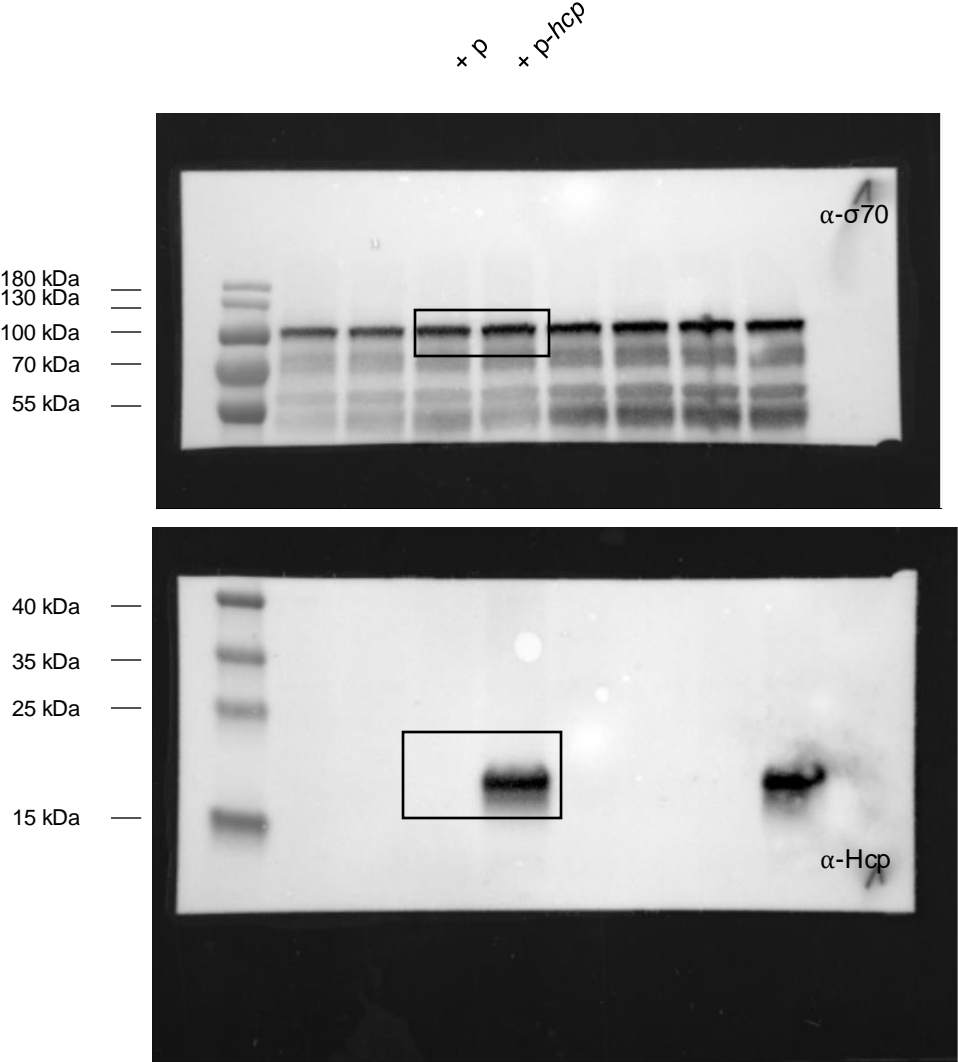

Supplement: Figure 7—figure supplement 1—source data 1. [file elife-101032-fig7-figsupp1-data1.pdf]

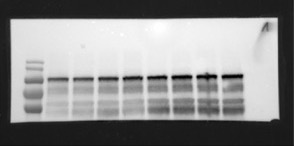

Supplement: Figure 7—figure supplement 1—source data 2. [file elife-101032-fig7-figsupp1-data2.zip › Sigma70.jpg]

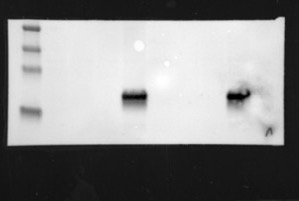

Supplement: Figure 7—figure supplement 1—source data 2. [file elife-101032-fig7-figsupp1-data2.zip › Hcp.jpg]

Figure 7-supplement 1B– source data-annotated

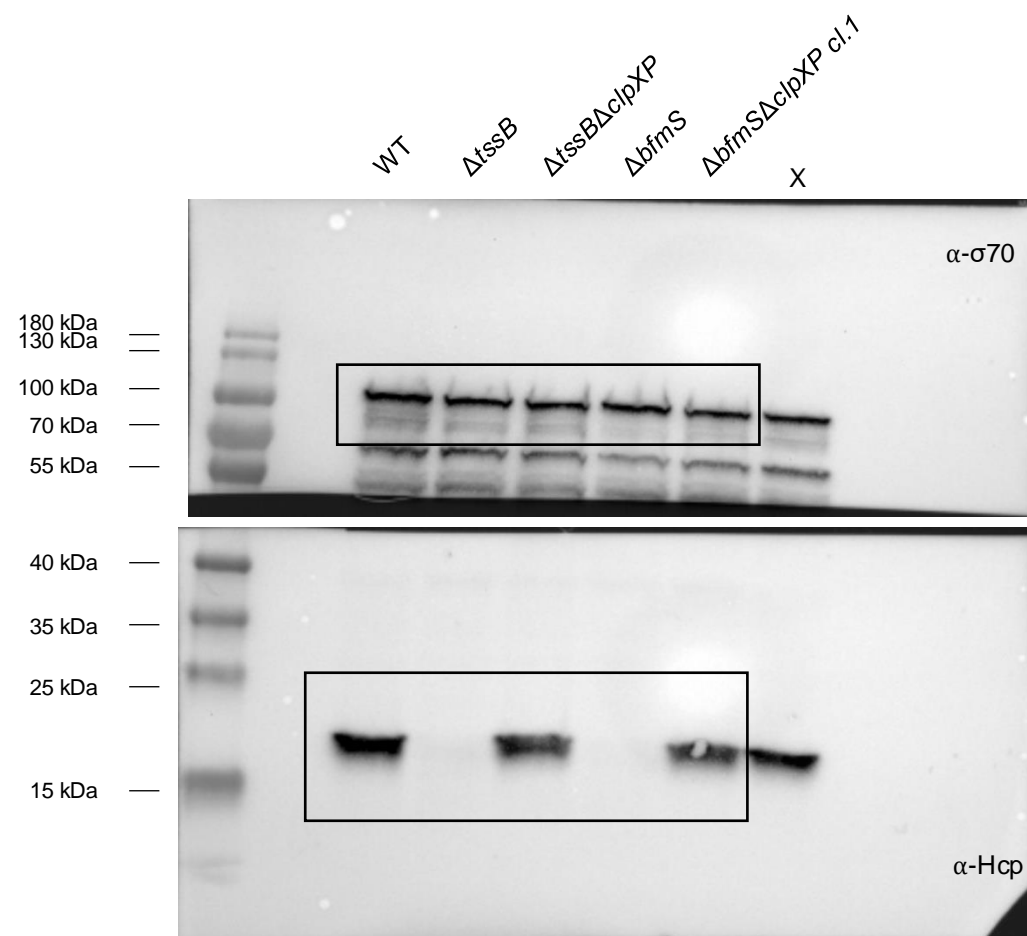

Supplement: Figure 7—figure supplement 1—source data 3. [file elife-101032-fig7-figsupp1-data3.pdf]

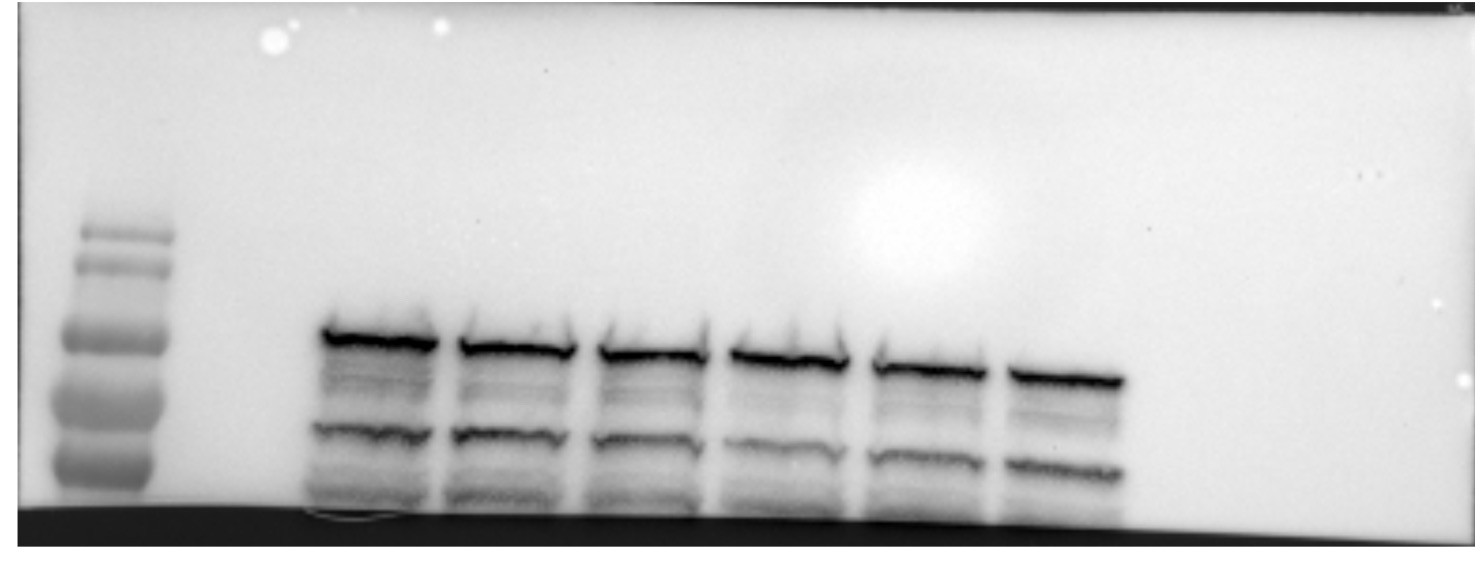

Supplement: Figure 7—figure supplement 1—source data 4. [file elife-101032-fig7-figsupp1-data4.zip › Sigma70.jpg]

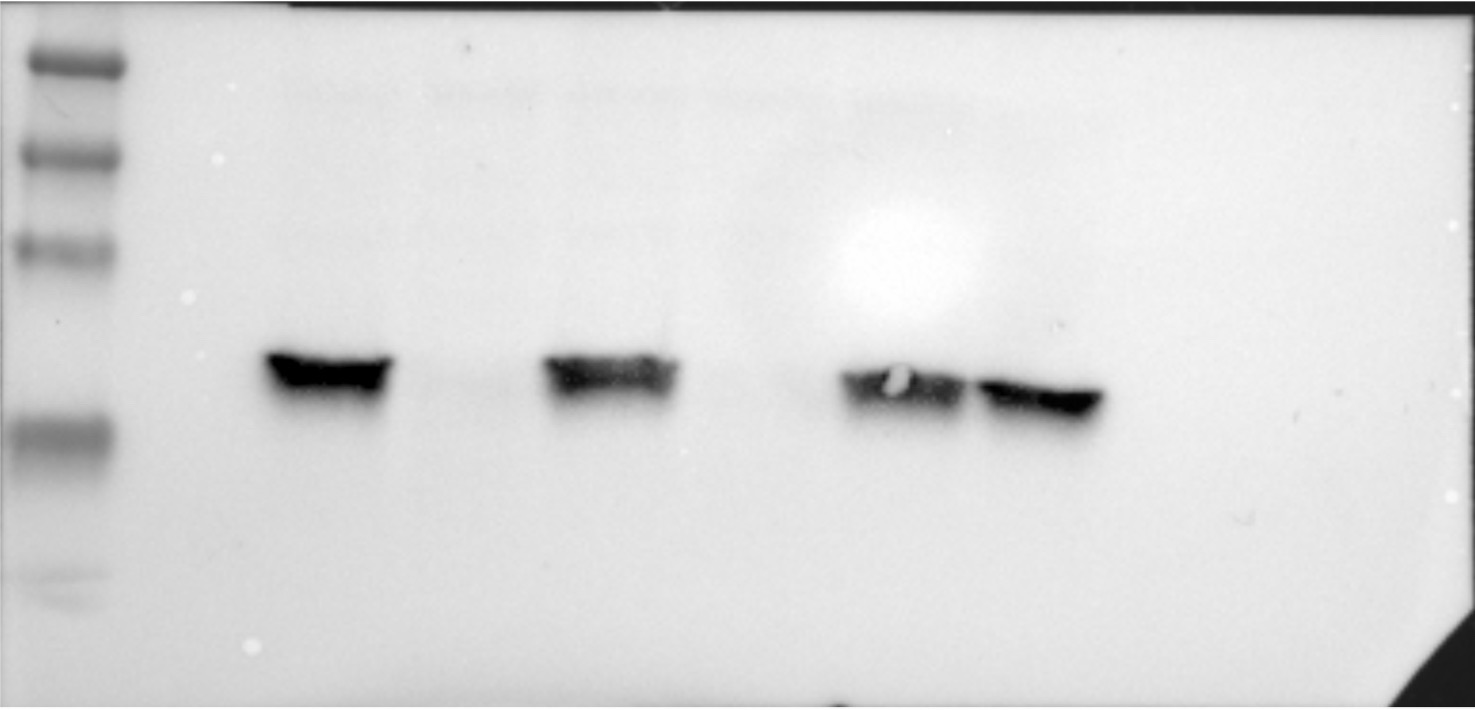

Supplement: Figure 7—figure supplement 1—source data 4. [file elife-101032-fig7-figsupp1-data4.zip › Hcp.jpg]

Figure 7-supplement 1E– source data-annotated

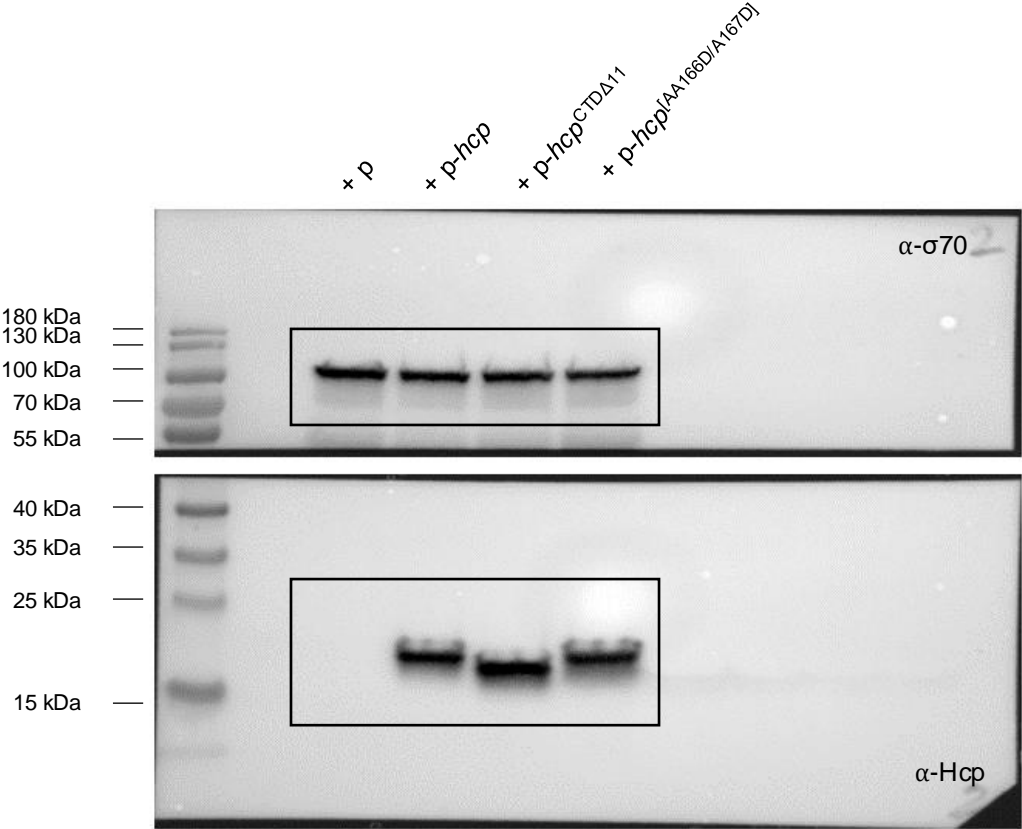

Supplement: Figure 7—figure supplement 1—source data 5. [file elife-101032-fig7-figsupp1-data5.pdf]

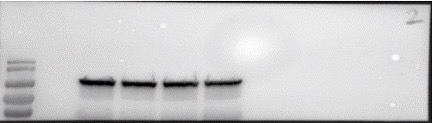

Supplement: Figure 7—figure supplement 1—source data 6. [file elife-101032-fig7-figsupp1-data6.zip › Sigma70.jpg]

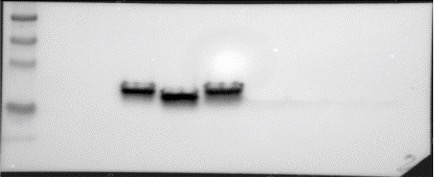

Supplement: Figure 7—figure supplement 1—source data 6. [file elife-101032-fig7-figsupp1-data6.zip › Hcp.jpg]
