# Supplementary material for: Capsular polysaccharide restrains type VI secretion in Acinetobacter baumannii
Source: eLife. 2025 Jan 3;14:e101032. doi: 10.7554/eLife.101032 (PMC11731876; doi:10.7554/eLife.101032)

Figure 6B – source data-annotated

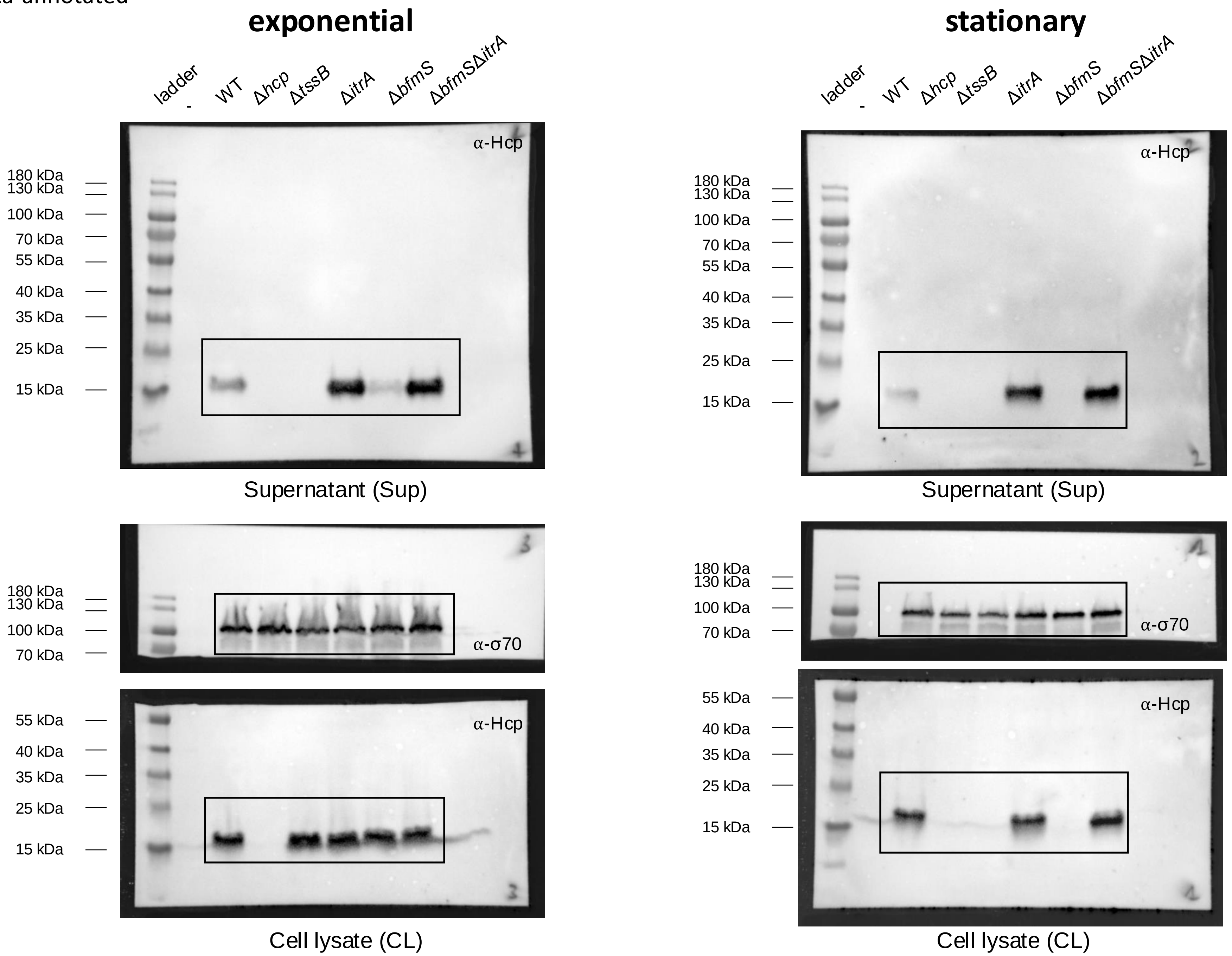

Supplement: Figure 6—source data 3. [file elife-101032-fig6-data3.pdf]
